# Surface Chemistry Engineering of Layered Double Hydroxides for Antigen Adsorption in Zika Virus Nanovaccine Development

**DOI:** 10.3390/nano16211419

**Published:** 2026-11-04

**Authors:** Sayma Adriana Rodriguez-Montelongo, Arleth Miranda-Lopez, Mariana Sanchez-Gil, Lourdes Betancourt-Mendiola, Veronica Perez-Luna, Mauricio Comas-Garcia, Mariano J. Garcia-Soto, Sergio Rosales-Mendoza, Omar Gonzalez-Ortega

**Affiliations:** 1 Facultad de Ciencias Químicas, Universidad Autónoma de San Luis Potosí, Av. Manuel Nava 6, San Luis Potosi 78210, Mexico; say.mont07@gmail.com (S.A.R.-M.); arlethmiranda@hotmail.com (A.M.-L.); a237003@alumnos.uaslp.mx (M.S.-G.); lourdes.betancourt@uaslp.mx (L.B.-M.); mariano.soto@uaslp.mx (M.J.G.-S.); 2 Sección de Biotecnología, Centro de Investigación en Ciencias de la Salud y Biomedicina, Universidad Autónoma de San Luis Potosí, Av. Sierra Leona 550, San Luis Potosi 78210, Mexico; 3 Sección de Microscopía de Alta Resolución, Centro de Investigación en Ciencias de la Salud y Biomedicina, Universidad Autónoma de San Luis Potosí, Av. Sierra Leona 550, San Luis Potosi 78210, Mexico; veronica.perez@uaslp.mx (V.P.-L.); mauricio.comas@uaslp.mx (M.C.-G.); 4 Facultad de Ciencias, Universidad Autónoma de San Luis Potosí, Av. Parque Chapultepec 1570, San Luis Potosi 78210, Mexico; 5 Department of Pharmacognosy and Pharmaceutical Botany, Faculty of Pharmaceutical Sciences, Chulalongkorn University, Bangkok 10330, Thailand

**Keywords:** amination, carboxylation, antigen adsorption, protein adsorption, immunogenicity, antigen delivery

## Abstract

Layered double hydroxides (LDH) are promising nanocarriers for vaccine delivery due to their biocompatibility and tunable surface properties. This study evaluated LDH and surface-functionalized derivatives containing amino (LDH–NH_2_) and carboxyl (LDH–COOH) groups as delivery platforms for a recombinant Zika virus (ZIKV) antigen (rpZDIII). The nanoparticles were synthesized by coprecipitation/hydrothermal treatment and afterward functionalized via silanization and succinylation. Their physicochemical characterization included hydrodynamic size, ζ potential, point of zero charge, and amine concentration, along with Fourier-transform infrared spectroscopy and thermogravimetric analyses. Protein adsorption was evaluated using bovine serum albumin, α-chymotrypsin, and the recombinant antigen rpZDIII. Cytotoxicity and immunogenicity were evaluated in HEK-293T cells and BALB/c mice, respectively. The functionalization affected the protein adsorption capacity and its retention after washing. The adsorption of rpZDIII was best described by the Freundlich model, with LDH–NH_2_ having the highest antigen adsorption and retention. All three formulations maintained high cell viability at lower nanoparticle concentrations (12.5–50 μg/mL). Immunization studies demonstrated that all nanoparticle formulations elicited strong antibody responses against rpZDIII; however, only unmodified LDH-induced antibodies were able to bind the full-length recombinant ZIKV envelope protein. These findings demonstrate that the surface chemistry of LDH influences the adsorption of antigens, their retention, and their further immune recognition, highlighting the importance of preserving antigen accessibility during the design of LDH-based nanovaccines.

## 1. Introduction

Layered double hydroxides (LDH) are synthetic inorganic clays composed of positively charged sheets with hydrated interlayer anions [1]. They are typically produced at the nanometer scale by precipitating soluble salts in a basic medium, followed by hydrothermal treatment [2]. Among their relevant properties for medical applications, LDH show biocompatibility and adjuvanticity [3]. Their natural positive charge makes them suitable for adsorbing negatively charged antigens in the development of subunit vaccines. In this regard, LDH have been evaluated against melanoma by using DNA [4], ovalbumin (OVA) [5], tyrosinase-related protein 2 [6], and neoepitopes (M27 and M30) [7] as antigens. LDH loaded with OVA and CpG has also been reported as a nanovaccine against lymphoma [8]. Additionally, LDH-based nanovaccines have been reported against bacterial pathogens such as *Escherichia coli* [9,10] and *Bordetella pertussis* [11], as well as viral pathogens such as porcine reproductive and respiratory syndrome virus (PRRSV) [12], severe acute respiratory syndrome coronavirus 2 (SARS-CoV-2) [13], porcine epidemic diarrhea virus (PEDV) [14], and foot-and-mouth disease virus (FMDV) [15]. Despite these reports, surface modification strategies aimed at enabling LDH to adsorb positively charged antigens, as well as the development of LDH-based nanovaccines against Zika virus, remain unexplored.

Due to the high density of surface hydroxyl (–OH) groups on LDH, organosilanes have been used for their functionalization. In this regard, Ádok-Sipiczki et al. [16] grafted (3-aminopropyl)triethoxysilane (APTES) onto the surface of LDH, obtaining a modified material capable of physically or chemically adsorbing a model 12-nucleotide-long molecule as inorganic carrier systems. Despite modifying the LDH surface, the material retained a positive charge, allowing the physical adsorption of a negatively charged molecule. In another study, Dinari & Neamati [17] used APTES-functionalized LDH as a support for aniline polymerization; the resulting composite was subsequently evaluated for Pb^2+^ removal. None of the previous studies explored organosilane-modified LDH for tuning antigen–surface interactions or for application in nanovaccine formulations.

Zika virus, an arbovirus of the *Flaviviridae* family, is responsible for Zika fever [18]. Although infections are often asymptomatic or have mild symptoms, during pregnancy an infection can result in microcephaly [19]. In adults, Zika virus infections have also been associated with Guillain–Barré syndrome [20]. As of 2025, no licensed vaccines are available to prevent Zika virus infections [21]. Nevertheless, two candidates have reached Phase II clinical trials, an mRNA-based vaccine (NCT04917861) [22] and a DNA-based vaccine (NCT03110770) [23], while other platforms (inactivated and viral-vector vaccines) have only reached Phase I [24].

Subunit vaccines against Zika virus have been investigated [25,26,27,28,29] due to their favorable safety profile and suitability for long-term storage and distribution [30]. Most subunit vaccines significantly increased the survival of mice challenged with the virus and were adjuvanted with Alum, AddaVax^®^ [31,32], CoVaccine HT^®^ [33,34], and monophosphoryl lipid A (MPLA) [35,36]. While some formulations elicited both humoral and cellular immune responses, others induced antibodies with varying degrees of cross-reactivity toward dengue virus [37,38]. Strategies aimed at targeted immune responses have also been pursued [39].

In this study, we report the development of a layered double hydroxide-based nanoplatform for antigen adsorption through surface chemistry engineering. Native and surface-modified LDH were first evaluated using two model proteins and subsequently combined with a recombinant EDIII-derived Zika virus protein fused to the B subunit of the heat-labile enterotoxin from *E. coli* (rpZDIII). The resulting antigen-loaded nanoparticles were physicochemically characterized, and their interaction with the two model proteins and with the recombinant antigen was systematically analyzed. The potential of these systems as nanovaccine candidates was further assessed through cytotoxicity and immunological evaluation.

## 2. Materials and Methods

### 2.1. Materials

Magnesium nitrate hexahydrate (Mg(NO_3_)_2_·6H_2_O, 99%), aluminum nitrate nonahydrate (Al(NO_3_)_3_·9H_2_O, ≥98%), (3-aminopropyl)triethoxysilane (APTES, 99%), glycine (≥98.5%), ninhydrin (≥99%), hydrindantin (≥97%), 2-amino-2-(hydroxymethyl)-1,3-propanediol (Trizma^®^ base, ≥99.9%), sodium dodecyl sulfate (SDS, ≥98.5%), polyethylene glycol sorbitan monolaurate (Tween^®^ 20), succinic anhydride (≥99%), acrylamide (≥99%), N,N′-methylenebisacrylamide (BIS, ≥99.5%), Coomassie Brilliant Blue R (≥70%), Coomassie Brilliant Blue G (≥80%), 2-mercaptoethanol (≥99%), ammonium persulfate (APS, ≥98%), N,N,N′,N′-tetramethylethylenediamine (TEMED, ≥99%), bovine serum albumin (BSA, ≥98%), and α-chymotrypsin from bovine pancreas (α-CHY, ≥85%; Type II, ≥40 units/mg protein) were purchased from Sigma-Aldrich (St. Louis, MO, USA).

Sodium acetate trihydrate (CH_3_COONa·3H_2_O, ≥99%) and sodium chloride (NaCl, ≥99%) were obtained from Vetec (Xerem, Brazil). Glacial acetic acid (≥99%) and hydrochloric acid (HCl, 37%) were purchased from BioChemsa (Tepotzotlan, Mexico) and Fermont (Monterrey, Mexico), respectively. Dimethyl sulfoxide (DMSO, ≥99%), sodium hydroxide (NaOH, ≥97%), and ethanol (≥99%) were supplied by Reproquifin (Xalostoc, Mexico). 12–14 kDa molecular weight cut-off (MWCO) Spectra/Por™ dialysis membranes were acquired from Repligen (Boston, MA, USA). Additionally, 0.1 µm pore-size Nuclepore™ polycarbonate filter disks were acquired from Whatman (Maidstone, UK).

### 2.2. Synthesis of Layered Double Hydroxides (LDH)

Layered double hydroxides (LDH) were synthesized following the coprecipitation protocol described by Xu et al. [40]. Briefly, 10 mL of an aqueous solution containing 0.15 M Mg(NO_3_)_2_·6H_2_O and 0.05 M Al(NO_3_)_3_·9H_2_O was rapidly added into 15 mL of 0.4 M NaOH under vigorous stirring using a Vortex-Genie^®^ 2 (Scientific Industries, Bohemia, NY, USA), keeping the precipitate formed under mixing for 10 min more. The resulting LDH precipitate was collected by two sequential centrifugation steps (10 and 30 min at 21,200× *g*) using a microcentrifuge Prism™ C2500 (Labnet, Edison, NJ, USA), with the supernatants discarded and the pellets redispersed in deionized water using a 750 W ultrasonic probe Sonics^®^ VCX 750 (Sonics & Materials, Newton, CT, USA) at 20% amplitude for 5 s.

The suspension was then transferred into a hermetically sealed PTFE-lined stainless-steel autoclave and subjected to hydrothermal treatment at 100 °C for 2 h in a Luzeren DHG-9240A drying oven (Qingdao, China). After cooling at room temperature, the product was recovered and washed by centrifugation (30 min, 21,200× *g*). The final LDH pellet was redispersed in either ethanol or deionized water (depending on subsequent functionalization or protein-loading steps) using the ultrasonic probe (20% amplitude, 5 s). The resulting suspension was characterized using dynamic light scattering (DLS) to determine the hydrodynamic diameter and polydispersity index (PDI) as described below. The LDH concentration was determined by centrifuging 1 mL of the suspension at 21,200× *g* for 30 min, discarding the supernatant, and placing the resulting pellet in a desiccator overnight to remove the residual solvent. The dried pellet was weighed using a Denver Instrument TP-64 analytical balance (Arvada, CO, USA), and the concentration was calculated as mg of solid per mL of suspension.

### 2.3. LDH Functionalization with Amino and Carboxylic Acid Groups

The LDH resuspended in ethanol were silanized by adding (3-aminopropyl)triethoxysilane (APTES) at a 50× molar excess to a 95% ethanol solution, followed by the immediate addition of LDH (2 mg/mL). Here, “×” denotes the calculated silane excess required to cover the surface of an LDH particle completely. The mixture was refluxed at 75 °C under continuous stirring for 2 h. Following the reaction time and two washing cycles (centrifugation at 21,200× *g* for 20 min and resuspension in ethanol), LDH particles with surface amino groups (LDH–NH_2_) were obtained in either deionized water or ethanol, depending on the subsequent functionalization step, at a final concentration of 2 mg/mL.

**Figure 1:**
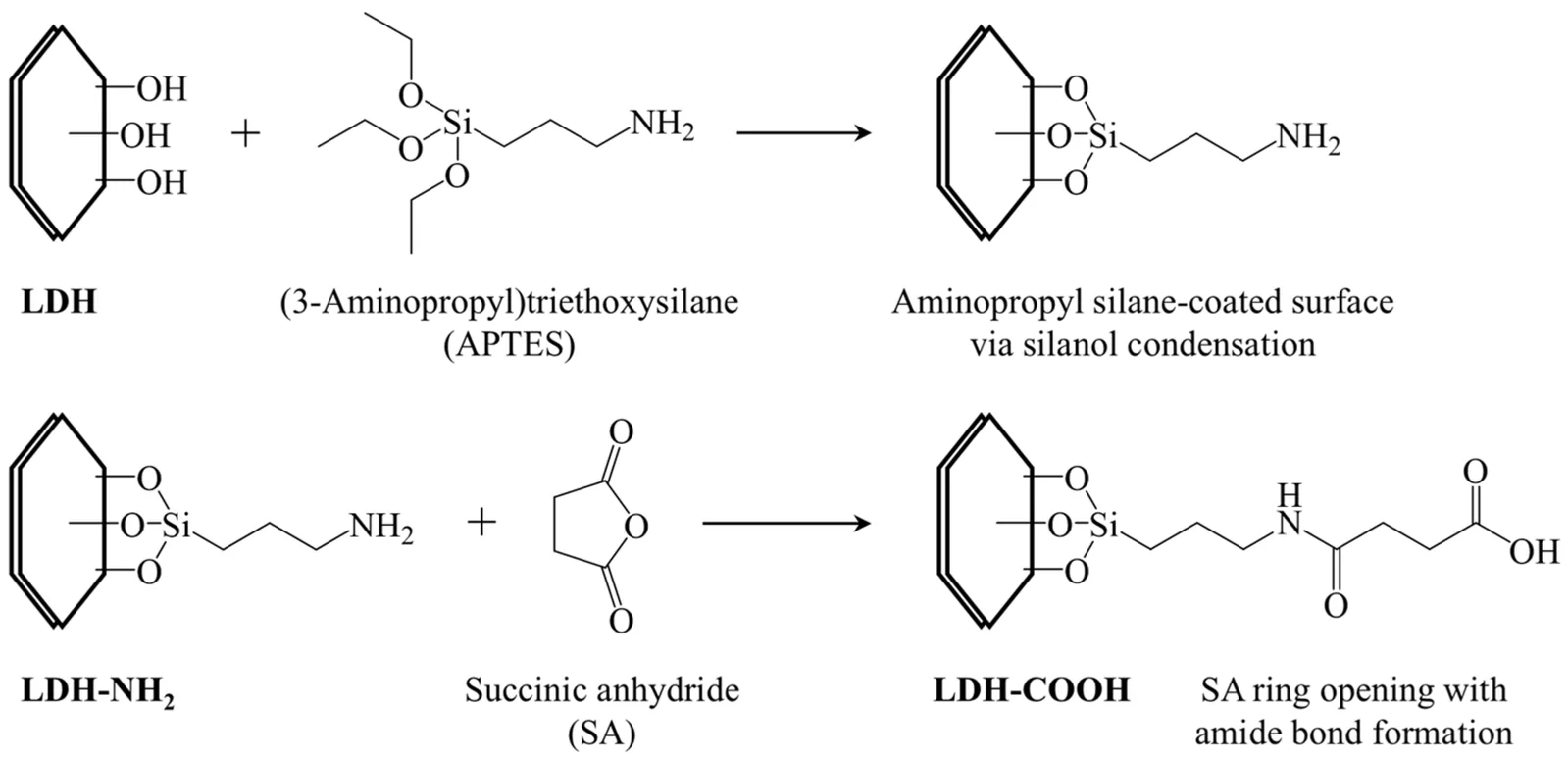
Functionalization of LDH via silanization with (3-aminopropyl)triethoxysilane (APTES), resulting in a covalent coating with primary amines, and their further succinylation due to the nucleophilic ring-opening of succinic anhydride (SA), leading to the amide bond formation between SA and surface amino groups on LDH.

Based on the ninhydrin assay (described in Section 2.6.1), the concentration of primary amino groups on the APTES-silanized particles (LDH–NH_2_) was quantified. This value was used to calculate the required concentration of succinic anhydride (SA) to form amide bonds with the primary amino groups available in LDH–NH_2_ and produce LDH with carboxylic groups (LDH–COOH). The functionalization of LDH-NH_2_ with SA proceeds via a nucleophilic acyl substitution reaction (Figure 1). The primary amino groups on the surface act as nucleophiles, attacking a carbonyl carbon of SA and inducing cleavage of the anhydride C–O bond. Afterward, a proton transfer process generates an amide bond between the surface aminopropyl groups and the succinyl moieties. Finally, the second carbonyl group forms the terminal carboxylic acid group.

### 2.4. Passive Adsorption with Model Proteins: BSA and α-CHY

Three types of particles (LDH, LDH–NH_2_, and LDH–COOH) were loaded with two model proteins: bovine serum albumin (BSA, 66 kDa), which has an isoelectric point (pI) of 5.1 and therefore a net negative charge at neutral pH [41], and bovine pancreatic chymotrypsin (α-CHY, 25 kDa), with a pI of 8.52 and a net positive charge at neutral pH [42].

The experiments were performed in deionized water in triplicate, wherein a fixed concentration of LDH, LDH–NH_2_, or LDH–COOH was contacted with BSA or α-CHY in different concentration ratios (Table 1) at room temperature and under continuous rotation for 1 h. After centrifuging at 21,200× *g* for 30 min, the supernatants were collected and filtered through a 0.1 µm pore-size membrane, and their absorbance was recorded at 280 nm. Protein concentrations at equilibrium in the liquid phase were determined using calibration lines prepared for both BSA and α-CHY with standard solutions of 25, 50, 100, 200, and 400 μg/mL. The equilibrium protein concentration in the solid phase was calculated by a mass balance, according to Equation (1).

(1) $$Q_{e}=\frac{C_{0}-C_{e}}{C_{\mathrm{ad}}}$$

where *Q_e_* is the equilibrium adsorption capacity, *C*_0_ is the initial protein concentration, *C_e_* is the equilibrium protein concentration measured in the supernatant, and *C_ad_* is the adsorbent concentration, i.e., the LDH nanoparticles. The Langmuir model was fitted to the experimental adsorption isotherm data for BSA and α-CHY to describe their adsorption behavior on LDH with different surface chemistries.

The resulting pellets were resuspended in deionized water and subjected to a second centrifugation–resuspension cycle under the same conditions as before. This washing procedure was relevant for evaluating the protein concentration that remained associated with the protein-loaded nanoparticles after removing the protein that was weakly bound. The protein concentration in the washed nanoparticles was analyzed by SDS-PAGE with a Mini-Protean Tetra Cell for vertical electrophoresis (Bio-Rad, Hercules, CA, USA) using BSA or α-CHY standards, followed by densitometric analysis with ImageJ 1.45s software (National Institutes of Health, Bethesda, MD, USA). The washed nanoparticles were further characterized by dynamic light scattering (DLS) as described below. The remaining protein concentration on the protein-loaded nanoparticles is denoted as *C_w_*.

### 2.5. Passive Adsorption with rpZDIII

Experiments with recombinant rpZDIII were performed in triplicate in Tris buffer (20 mM, pH 8.1) containing 10% sucrose and 0.01% Tween 20, using fixed particle concentrations (LDH, LDH–NH_2_, or LDH–COOH) and increasing concentrations of the recombinant protein, according to the same ratios listed in Table 1.

The adsorption was carried out at room temperature under continuous rotation for 1 h. The antigen-loaded nanoparticles were subsequently subjected to two washing cycles (centrifugation at 21,200× *g* for 30 min) followed by redispersion in a Branson CPX1800H (40 kHz) ultrasonic bath (Danbury, CT, USA) using the same Tris buffer and finally resuspended in 10% sucrose with 0.01% Tween 20 medium. The supernatants collected after the first washing cycle were retained for subsequent UV–Vis spectroscopic analysis after filtering them through a 0.1 µm pore-size membrane. After the second washing step, the protein concentration in the resulting nanoparticles was analyzed by SDS-PAGE.

Adsorption isotherms for the recombinant rpZDIII protein were obtained following a procedure similar to that described for the model proteins. The Freundlich model was subsequently fitted to the experimental isotherm data. The rpZDIII concentration that remained associated with the nanoparticles after the second washing is denoted as *C_w_*.

### 2.6. Physicochemical Characterization

Pellets of LDH, LDH–NH_2_, LDH–COOH, and LDH–NH_2_–rpZDIII were recovered by centrifugation at 21,200× *g* for 30 min. The supernatants were discarded, and the pellets were dried overnight in a vacuum desiccator to remove any residual solvent.

In parallel, rpZDIII in 10% sucrose with 0.01% Tween 20 was precipitated by the slow addition of acetone previously cooled to −20 °C. The suspension was maintained at −20 °C for 1 h, then centrifuged at 15,200× *g* for 1 min. The supernatant was discarded, and the resulting pellet was placed in a vacuum desiccator and dried overnight.

All samples were characterized by Fourier-transform infrared (FT-IR) spectroscopy using the attenuated total reflectance (ATR) technique with a Thermo Scientific Nicolet iS 10 FTIR spectrometer (Waltham, MA, USA). The spectra were recorded in the 4000–400 cm^−1^ range, in transmittance mode, with a spectral resolution of 8 cm^−1^ and 32 scans per sample.

Subsequently, all samples were characterized by thermogravimetric analysis (TGA) using a TA Instruments Waters Discovery TGA 550 (New Castle, DE, USA) under nitrogen at a flow rate of 60 mL/min. The samples were placed in a platinum HT pan and heated from an initial equilibrium temperature of 30 °C to 1000 °C at a heating rate of 10 °C/min.

The hydrodynamic diameter, polydispersity index (PDI), and ζ potential were determined by dynamic light scattering (DLS) using a Malvern Panalytical Zetasizer Pro (Malvern, UK). Water was declared as the dispersant and MgAl_2_O_4_ was selected as the dispersed material, with refractive indices of 1.33 and 1.71, respectively. For ζ potential measurements, the samples were diluted 1:10 and supplemented with 1 mM NaCl to increase ionic strength [43]. The size and morphology of the LDH particles were characterized by transmission electron microscopy (TEM) using a JEOL JEM-2100 microscope (Akishima, Japan) operated at 200 kV. High resolution regions were analyzed by fast Fourier transform (FFT), using the Gatan DigitalMicrograph software (version 2.30.542.0) to confirm the crystalline domains of the synthesized LDH.

The measurement media and experimental conditions for colloidal characterization are specified for each technique, with experiments performed in triplicate. The remaining physicochemical characterization analyses were performed on single samples.

#### 2.6.1. Ninhydrin Assay

The chemical modifications of the surface-functionalized LDH were monitored by quantifying primary amine groups using the ninhydrin assay, adapting the original protocol developed by Moore & Stein [44]. This assay involved preparing fresh calibration lines of increasing glycine (Gly) concentrations from 0 to 100 µM. Simultaneously, a reagent solution composed of 2% ninhydrin and 0.3% hydrindantin in a solvent mixture of 75% DMSO and 25% sodium acetate buffer (4 M, pH 5.5) was prepared and referred to as solution A. The assay was performed by adding solution A to the samples of interest (LDH, LDH–NH_2_, or LDH–COOH) and to the Gly standards at a 1:0.5 ratio. The samples and standards were incubated in a Labnet AccuBlock™ D1100 Digital Dry bath (Edison, NJ, USA) at 100 °C for 15 min. After heating, deionized water was added to each sample at a 1:1 volume ratio, and the absorbance was measured immediately at 570 nm using a Thermo Scientific Genesys™ 50 UV–Vis spectrophotometer (Waltham, MA, USA). The analysis was performed in three independent experiments.

#### 2.6.2. Point of Zero Charge

The point of zero charge (PZC) was determined using the pH-drift method in the presence of a background electrolyte [45]. A 0.01 M NaCl solution was prepared as the supporting electrolyte and subsequently degassed under vacuum to minimize interference from dissolved CO_2_. Aliquots of 25 mL of the degassed electrolyte were transferred into separate containers, and the initial pH (pH*_i_*) was adjusted within the range of 3–12 using HCl and NaOH solutions of appropriate concentrations. Following the pH adjustment, 2 mL of each solution was transferred into glass test tubes.

The particles were previously degassed by immersion in an ultrasonic bath for 5 min to reduce dissolved gases and potential surface adsorption effects. The material was then added to each tube to reach a final concentration of 0.1 mg/mL. The suspensions were homogenized with a vortex mixer and immediately sealed to prevent exchange with atmospheric CO_2_. The systems were allowed to equilibrate for 48 h at room temperature. After equilibration, the final pH (pH*_f_*) of each suspension was measured using a Bante Instruments 920 pH meter (Shanghai, China) with a Hanna Instruments HI1332B pH electrode (Smithfield, RI, USA).

The procedure was performed separately for as-prepared LDH, amino-functionalized LDH (LDH–NH_2_), and carboxyl-functionalized LDH (LDH–COOH) samples to evaluate the influence of their surface modification on the protonation–deprotonation behavior. The PZC was determined graphically by plotting pH*_f_* versus pH*_i_*. The point at which these two values are equal defines the point of zero charge. The analysis was performed in three independent experiments.

#### 2.6.3. Cytotoxicity Assessment

The cytotoxicity of LDH, LDH–NH_2_, and LDH–COOH was evaluated using HEK-293T cells. These cells were maintained in Gibco™ Dulbecco’s Modified Eagle’s Medium (DMEM) supplemented with Biowest 10% fetal bovine serum (Bradenton, FL, USA) and 1% penicillin–streptomycin solution (100×, Sigma-Aldrich) under humidified conditions at 37 °C and 5% CO_2_.

The assay started with 1 × 10^4^ cells per well, seeded in triplicate in 96-well culture plates and allowed to adhere for 24 h. Afterward, the cells were exposed to different nanoparticle concentrations (10–250 µg/mL) for 48 or 72 h. Control groups included cells treated with the vehicle alone (DMEM) as negative control or H_2_O_2_ (40 mM) as a positive cytotoxicity control.

The cell viability was determined using the resazurin assay. After each treatment, the cells were incubated with resazurin solution (30 µg/mL) for 4 h, and fluorescence was measured at excitation/emission wavelengths of 560/590 nm using a BioTek Instruments Cytation™ Cell Imaging Multi-Mode Reader (Winooski, VT, USA). Measurements were performed in triplicate for each sample.

### 2.7. Immunization Assessment

Mice (twelve-week-old BALB/c) were maintained under controlled temperature and environmental conditions. All experimental procedures were approved by the Institutional Ethics Committee (CEID-2015-069). The nanovaccines were generated by passive adsorption of rpZDIII at a 1:2 particle-to-antigen ratio, with a fixed particle concentration of 200 μg/mL. The resulting antigen-loaded nanoparticles were washed twice through centrifugation (12,200× *g*, 30 min) and resuspension cycles to remove weakly bound protein. Afterward, the concentration of the remaining antigen adsorbed was quantified by SDS-PAGE. Antigen retention was calculated as the percentage of protein remaining after washing relative to the concentration before washing. The resulting washed suspensions were diluted to a final antigen concentration of 25 μg/mL for immunization. To reduce the estimated osmolality of the final formulations, the 20 mM Tris buffer was omitted when preparing the nanovaccines. The final nanovaccines were suspended in a vehicle containing 10% sucrose and 0.01% Tween 20. The corresponding nanoparticle concentrations were calculated by dividing the initial particle concentration (200 μg/mL) by the dilution factor used to obtain the target antigen concentration. The three formulations were prepared in a single experiment.

Mice were randomly distributed in experimental groups (*n* = 5 per group) and immunized subcutaneously (s.c.). Each animal received three 200 µL-doses (each containing 5 µg of rpZDIII) on days 0, 14, and 28. Mice were administered one of the following formulations: vehicle (10% sucrose with 0.01% Tween 20, as negative control), LDH-rpZDIII, LDH–NH_2_-rpZDIII, or LDH–COOH-rpZDIII. Blood samples were collected via tail puncture on days 27, 42, and 56. Serum samples were obtained by centrifugation at 6000 rpm for 10 min and stored at −40 °C until antibody analysis.

### 2.8. Determination of Antibody Levels by Enzyme-Linked Immunosorbent Assay (ELISA)

Antigen-specific IgG antibody levels were determined by indirect ELISA using two target antigens: the recombinant rpZDIII protein and recombinant ZIKV envelope (E) protein (Cat. No. 40543-V08B4, Sino Biological, Beijing, China). Ninety-six-well polystyrene microplates were coated overnight at 4 °C with 200 ng/well of antigen diluted in carbonate buffer (pH 9.6). Plates were subsequently blocked with 5% fat-free powdered milk suspension for 2 h at room temperature.

Serum samples were added to the plates with one measurement per animal (1:3000 dilution for rpZDIII and 1:100 dilution for ZIKV E protein) and incubated overnight at 4 °C. Afterward, goat anti-mouse IgG conjugated to horseradish peroxidase (HRP) (1:2000 dilution) was added as the secondary antibody and incubated for 2 h at room temperature. Antibody binding was visualized using an ABTS substrate solution. Following a 60 min incubation at 25 °C, optical density (OD) was measured at 405 nm using a Multiskan™ FC microplate photometer (Thermo Fisher Scientific, Waltham, MA, USA). Prior to each incubation step, plates were washed three times with PBST (PBS containing 0.05% Tween 20).

### 2.9. Statistical Analyses

All experiments were performed in triplicate unless otherwise stated, and the results are presented as mean ± standard deviation. Statistical analyses were carried out using one-way analysis of variance (ANOVA) to evaluate differences among experimental groups. When significant differences were detected, multiple-comparison analyses were performed using Tukey’s post hoc test for pairwise comparisons among all groups or Dunnett’s post hoc test when comparing multiple treatments against a control group. Differences were considered statistically significant at *p* < 0.05. When assessing cytotoxicity at different nanoparticle concentrations and at two different time points, a two-factor ANOVA analysis was applied.

## 3. Results and Discussion

### 3.1. LDH Synthesized and Functionalized as LDH–NH_2_, and LDH–COOH

#### 3.1.1. Hydrodynamic Size, PDI, and ζ Potential

Freshly synthesized LDH obtained by coprecipitation/hydrothermal treatment consistently had a hydrodynamic diameter of 70.3 ± 1.7 nm and a PDI of 0.133 ± 0.021, indicating a monodisperse particle distribution (Figure S1). A TEM image shows the morphology of LDH in Figure S1. Moreover, the analysis of high-resolution TEM images confirms the crystalline domains of Mg-Al LDH (Figure S2).

After their silanization, rendering LDH–NH_2_, the particle size increased to 109.5 ± 6.6 nm, consistent with the incorporation of organic moieties onto the surface [46], with a PDI of 0.078 ± 0.070. Their further functionalization with succinic anhydride resulted in LDH–COOH with an additional increase in size to 152.4 ± 5.3 nm and a PDI of 0.090 ± 0.022. In all cases, the PDI values below 0.3 confirm a high degree of colloidal homogeneity [47].

Regarding the ζ potential, the non-functionalized LDH had a positive value of +50.5 ± 5.7 mV. After silanization, the ζ potential as LDH–NH_2_ decreased to +36.6 ± 1.3 mV, attributed to the formation of covalent bonds with surface hydroxyl groups and additional non-covalent interactions involving amine functionalities, leading to a reduction in the effective density of positively charged sites at the interface [48,49].

Following their functionalization with succinic anhydride, rendering LDH–COOH, a shift in ζ potential toward negative values (−24 ± 3.3 mV) was observed due to the introduction of carboxylic groups. However, the magnitude of this negative charge is attenuated by the presence of residual amine groups and the intrinsic positive charge of the LDH layers, which arises from structural substitutions and lattice disorder. Additionally, the ionic species present in the aqueous medium contribute to interfacial charge regulation. As a result, the overall surface charge reflects a heterogeneous distribution governed by the coexistence of amine and carboxyl functionalities rather than a purely negative surface.

#### 3.1.2. Concentration of Amino Groups

Primary amine groups on LDH were quantified using the ninhydrin assay after silanization with APTES and subsequent functionalization with succinic anhydride (SA). After silanization with APTES, the amine content increases significantly to 597.2 ± 39.6 µM at a particle concentration of 2 mg/mL. Following functionalization with SA, the concentration of primary amines decreases markedly to approximately 25 ± 7.2 µM, corresponding to ~95.8% conversion of surface amino groups consumed during the succinylation reaction, confirming the efficiency of the carboxylation process. Despite this high conversion, a residual fraction of amino groups remained, attributable to steric hindrance and heterogeneous surface accessibility. These remaining amine functionalities are relevant for understanding the surface charge behavior and adsorption properties discussed in subsequent sections, since they contribute to the amphoteric character of the LDH surface.

#### 3.1.3. Point of Zero Charge (PZC)

**Figure 2:**
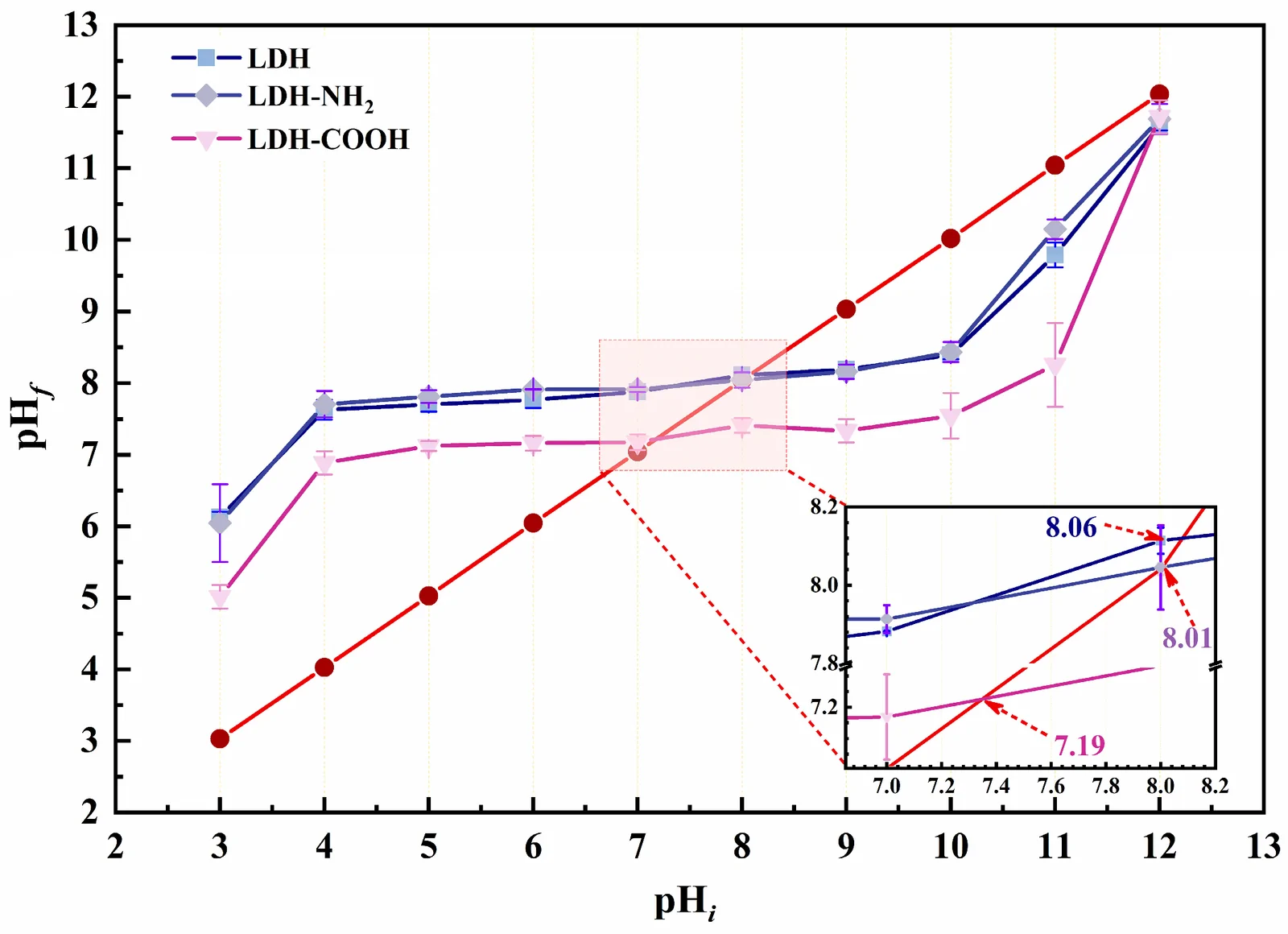
PZC of LDH, LDH–NH_2_, and LDH–COOH, determined from the intersection between the initially set pH (pH*_i_*) and the pH measured after 48 h (pH*_f_*). The red line represents the reference (pH*_f_* = pH*_i_*), while the red dashed lines indicate the region magnified in the inset. LDH–COOH shows a shift toward lower pH compared with LDH and LDH–NH_2_. Data are presented as average ± standard deviation (SD) from three independent experiments.

The PZC was determined from the intersection of the measured pH values with the initial pH of the suspensions, which were allowed to equilibrate for 48 h until a stable pH was reached. LDH had a PZC of 8.06, while LDH–NH_2_ and LDH–COOH showed PZC values of 8.01 and 7.19, respectively (Figure 2). Notably, the PZC of LDH–NH_2_ did not significantly differ from that of LDH. This behavior can be attributed to the consumption of surface hydroxyl groups during silanization, which is partially compensated by the introduction of amine functionalities via APTES, as well as the intrinsically positive character of the LDH layers. This observation is consistent with the ζ potential measurements, which showed no increase in surface positivity for LDH–NH_2_ relative to LDH; instead, a slight decrease in surface charge was observed.

For LDH–COOH, the introduction of surface carboxylate groups provided an anionic contribution that shifts the PZC toward more acidic pH values, as a higher degree of protonation is required to achieve charge neutrality [50]. However, this shift was moderate, attributable to residual surface amino groups and the intrinsic positive charge of the LDH layers, resulting in heterogeneous acid-base surface behavior.

These variations in surface charge cannot be attributed to a single functional group, but rather to the distribution of multiple surface species and their coordination with the LDH lattice, which are governed by the crystal structure and surface composition. Accordingly, the effective charge of surface functional groups depends on the degree of neutralization by the metal ions within the LDH structure [51].

Furthermore, the pKa values of amino and carboxyl groups differ significantly from those observed in solution when these functionalities are anchored on a surface. This effect is due to the local electrostatic environment, the interactions with neighboring functional groups, and the LDH intrinsic charge. As a result, the apparent pKa values in these systems are governed by the interfacial pH and electrostatic effects rather than their intrinsic values in solution, a phenomenon commonly referred to as an effective pKa. Consequently, the PZC reflects a collective surface charge regulation process, which explains the moderate shift observed and its deviation from the behavior expected for isolated functional groups [52].

#### 3.1.4. Cytotoxicity Assessment

**Figure 3:**
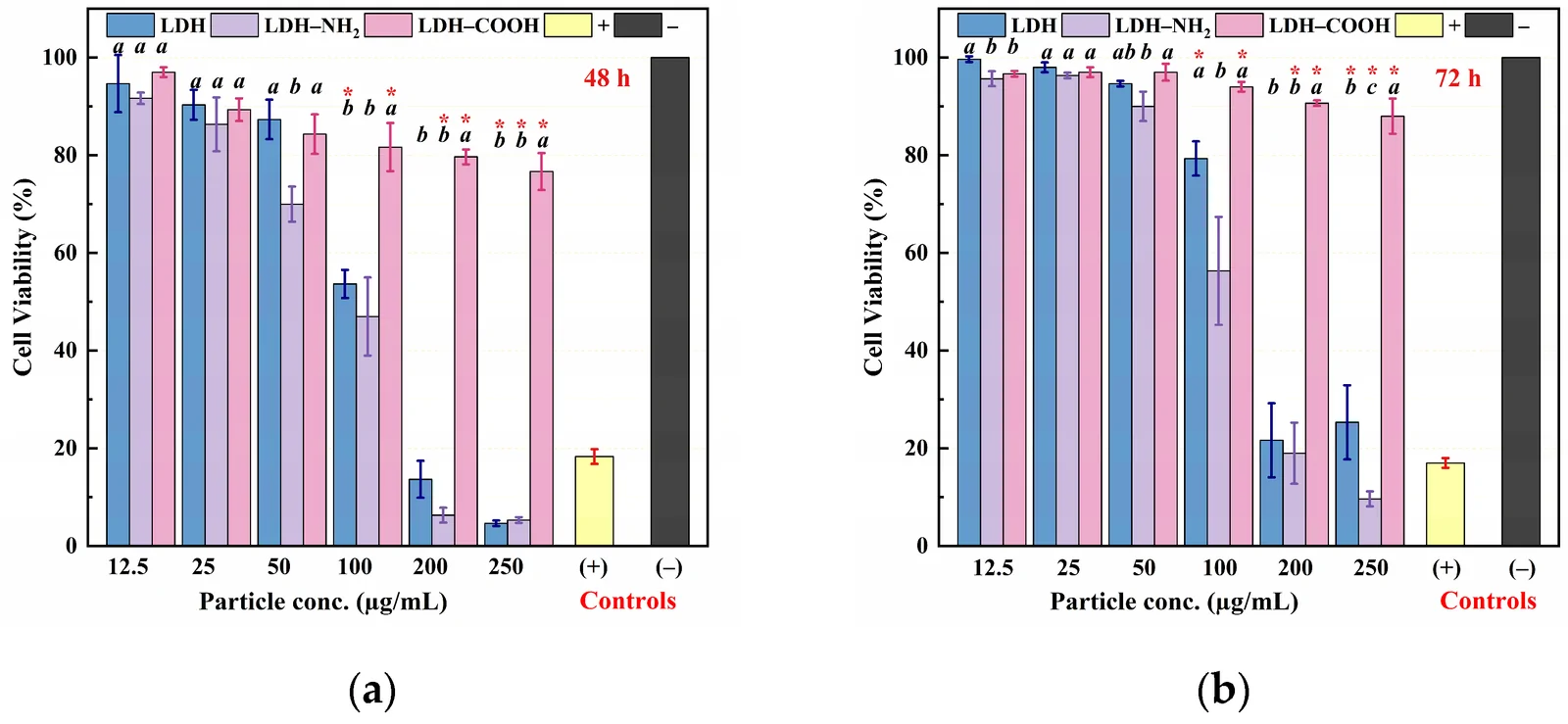
Cytotoxicity assessment with increasing concentrations (12.5–250 μg/mL) of LDH, LDH–NH_2_, and LDH–COOH added to HEK-293T cell cultures at (**a**) 48 h and (**b**) 72 h. H_2_O_2_ (40 mM) and DMEM were used as positive and negative controls, respectively. Different letters represent statistically significant differences (*p* < 0.05) comparing all particles at the same concentration, while the red asterisks represent treatments in which the cell viability increased significantly from 48 to 72 h. Data are presented as average ± standard deviation (SD) of three technical replicates per condition.

The cytotoxicity results show that the cell viability at 48 and 72 h strongly depended on both the nanoparticle concentration and their surface functionalization, as validated by a two-factor ANOVA analysis. Furthermore, the analysis revealed a statistically significant interaction between particle type and concentration, showing that the effect of concentration on cell viability varied according to nanoparticle surface chemistry. At low concentrations (12.5–50 µg/mL), all LDH formulations (functionalized or not) exhibited high cell viability (>85%), indicative of minimal toxicity under these conditions. However, as the concentration increased, noticeable differences emerged among the materials. LDH and LDH–NH_2_ caused a substantial reduction in viability at concentrations ≥ 100 µg/mL after 48 h of exposure. In contrast, LDH–COOH maintained high viability even at the highest concentrations tested, preserving approximately 75–90% viability after both 48 and 72 h. These results indicate that surface functionalization affected the in vitro cytotoxicity profile of the LDH nanoparticles under the conditions studied. LDH–COOH maintained higher cell viability at the concentrations evaluated, while LDH and LDH–NH_2_ showed greater reductions in viability at higher concentrations. These differences may be related to changes in the particle surface properties; however, the specific mechanisms responsible for the cytotoxic responses observed were not studied in this work. Moreover, an ANOVA analysis demonstrated the effect of exposure time on cell viability at toxic concentrations of 100, 200, and 250 µg/mL. In most particle-type-to-concentration combinations (Figure 3), the cell viability showed a significant increase between 48 and 72 h, indicating a time-dependent cytotoxic response. However, this trend was not observed for LDH at 200 µg/mL and LDH–NH_2_ at 100 µg/mL, where viability remained statistically unchanged between time points.

It is worth mentioning that the nanoparticle concentration employed in the nanovaccine formulation (<50 µg/mL) falls within a concentration range in which all LDH formulations preserved high cellular viability (>85%) after both 48 and 72 h incubation.

### 3.2. Protein Adsorption Studies

#### 3.2.1. Adsorption of Model Proteins BSA and α-CHY

Exploratory adsorption studies were performed using LDH particles with three different surface chemistries (–OH, –NH_2_, –COOH) and two model proteins (BSA and α-CHY) to evaluate the influence of the surface functional groups on the particles and their protein loading capacity. The experimental data were fitted to the Langmuir model *Q_e_* = *Q_m_C_e_*/(*K_L_* + *C_e_*) (Figure S3), and the fitting parameters and goodness-of-fit statistics are presented in Table S1. For BSA, the unmodified LDH showed the highest maximum capacity (*Q_m_*), while LDH–NH_2_ showed the lowest *K_L_* value, indicating that a lower equilibrium concentration was required to reach half of the fitted *Q_m_* value. In contrast, carboxylation reduced the adsorption capacity and the apparent affinity for BSA. For α-CHY, LDH–COOH showed the highest adsorption capacity and the lowest *K_L_* value, which suggests a more favorable adsorption under the studied conditions. Even though the Langmuir model provided an empirical description of the isotherms measured, the fitting alone does not establish if the adsorption process occurs as a monolayer or if a single class of adsorption site exists. Electrostatic complementarity may contribute to the differences observed, considering the net charge of the proteins and the surface properties of the nanomaterials. Despite this, α-CHY still became adsorbed onto both LDH and LDH–NH_2_, which suggests that other mechanisms, such as hydrogen bonding and van der Waals forces, could also participate in the adsorption process. These possible interactions were not quantified in this study.

The analysis of the protein that remained bound to the nanoparticles after two washing cycles revealed that BSA was still retained on LDH and LDH–NH_2_ (Figure S3), while LDH–COOH showed negligible adsorption. These results imply that the interaction between BSA and LDH–COOH was relatively weak so that it was effectively disrupted during the washing procedure. Conversely, α-CHY showed an opposite behavior, remaining bound only to LDH–COOH after the same washing cycles (Figure S3), while a negligible amount of protein was detected on both LDH and LDH–NH_2_. These results indicate that the surface functionalization of LDH not only influences the extent of protein adsorption but also affects the strength of protein–nanoparticle interactions. This aspect is relevant for nanovaccine development, where sufficient antigen retention in the carrier is required to ensure efficient delivery to antigen-presenting cells. If the antigen is rapidly released from the nanoparticle before the cellular uptake, the advantages of nanoparticle-mediated delivery may be compromised, potentially resulting in a weaker immune response.

The hydrodynamic size measurements showed that at low BSA concentrations, the protein-loaded nanoparticles remained below 200 nm and had PDI values below 0.2, indicating good colloidal dispersity in suspension. However, a marked increase in particle size was observed for LDH–BSA and LDH–NH_2_–BSA at intermediate particle-to-protein ratios (1:0.5), suggesting the existence of a partial surface coverage that promotes interparticle bridging through protein-particle and protein–protein interactions. At higher protein concentrations, the subsequent reduction in size suggests improved steric stabilization resulting from increased surface coverage. In contrast, the nanoparticles loaded with α-CHY remained comparatively stable, likely due to the smaller and more compact structure of the protein, which may reduce bridging effects and favor the formation of more homogeneous adsorption layers (Figure S4).

Systems exhibiting limited protein adsorption showed only minor variations in ζ potential, suggesting weak interfacial interactions and low surface coverage. In contrast, systems exhibiting favorable adsorption showed progressive shifts in ζ potential that were proportional to the increase in protein concentration, reflecting the gradual modification of the particle surface by the adsorbed protein layer. This behavior was particularly evident in electrostatically favorable systems, where increasing protein adsorption progressively altered the interfacial charge environment, while non-favorable systems remained comparatively stable due to the limited amount of adsorbed protein.

Although BSA and α-CHY possess hydrophobic domains and heterogeneous charge distributions, their adsorption behavior under the evaluated conditions appeared to be influenced by electrostatic complementarity with the particle surface. These results suggest that surface functionalization strongly influences the protein’s adsorption behavior and can modulate interfacial stability depending on the physicochemical properties of the biomolecule. In addition, the secondary structure of BSA is predominantly composed of α-helices (~67%) [53], which contributes to its conformational stability through extensive non-covalent intramolecular interactions that reinforce protein folding and structural robustness [54]. In contrast, α-CHY consists primarily of antiparallel β-pleated sheets forming short irregular strands organized into two antiparallel β-barrel domains composed of a Greek-key motif followed by an antiparallel hairpin motif with a minor α-helical contribution. Furthermore, the structural stability of α-CHY is reinforced by five disulfide bridges connecting its three polypeptide chains, together with its compact globular structure (~4.4 nm) [55].

Proteins adsorb rapidly during the initial stages of interaction, followed by a gradual decrease in the adsorption rate as the surface coverage increases. Due to their globular conformation, both BSA and α-CHY tend to adsorb predominantly in monolayers regardless of their orientation upon interaction with the surface. In contrast, multilayer adsorption is typically associated with molecular rearrangement processes in which the protein orientation enhances hydrophobic intermolecular interactions while simultaneously allowing additional molecules to adsorb onto available surface sites. This type of multilayer adsorption is observed at high protein concentrations [56].

#### 3.2.2. Adsorption of the Recombinant Protein rpZDIII

**Figure 4:**
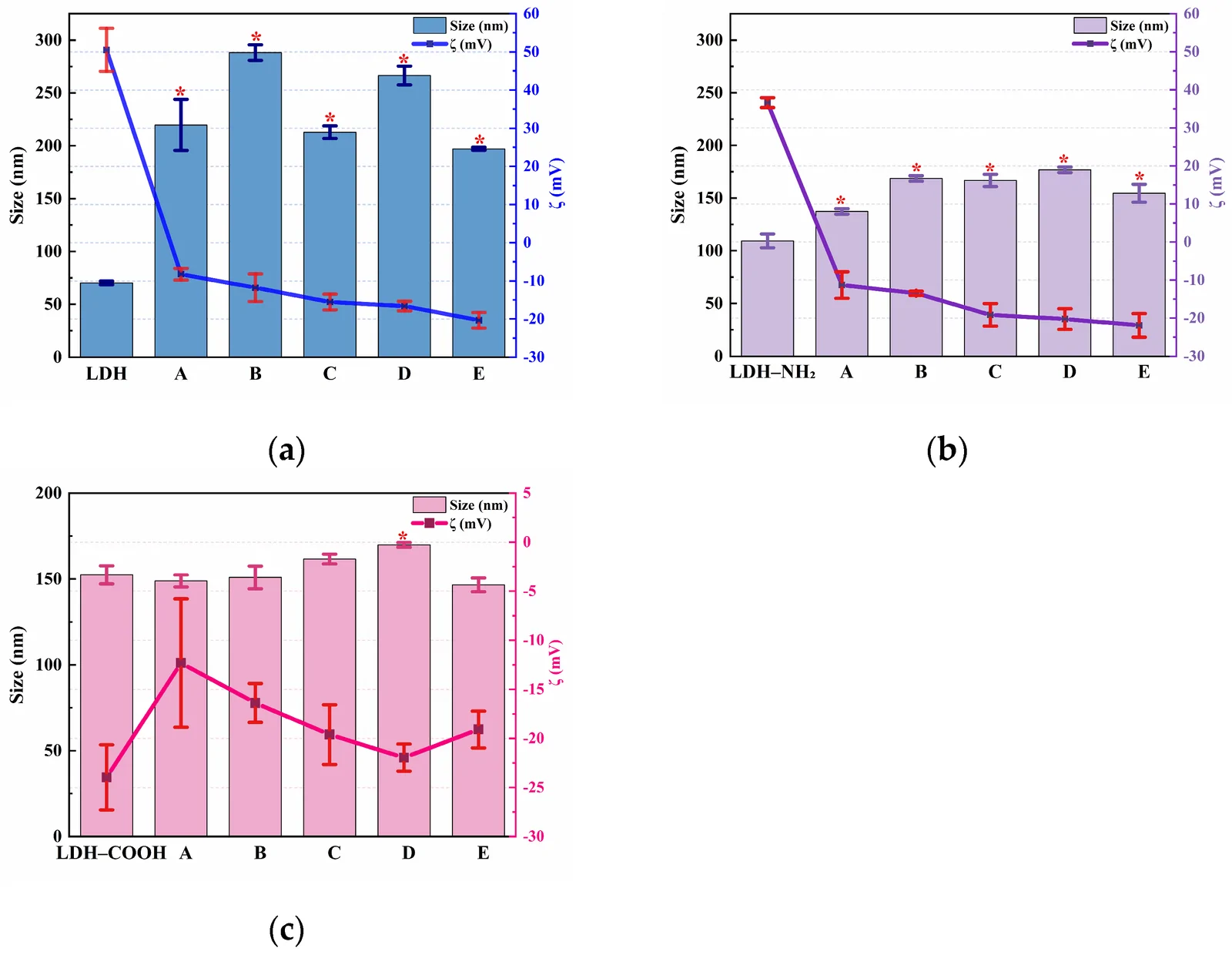
Hydrodynamic size (bars) and ζ potential (lines) of (**a**) LDH, (**b**) LDH–NH_2_, and (**c**) LDH–COOH having adsorbed the recombinant protein rpZDIII at different particle-to-protein ratios (A–E corresponding to 1:0.125, 1:0.25, 1:0.5, 1:1, and 1:2 ratios, respectively). Data are presented as average ± standard deviation (SD) from three independent experiments. Asterisks represent statistically significant differences (*p* < 0.05) comparing the size of the A–E treatments with the control nanoparticles (LDH, LDH–NH_2_, or LDH–COOH).

Upon passive adsorption with rpZDIII at different particle-to-protein ratios, the unmodified LDH had a pronounced increase in hydrodynamic diameter, while LDH–NH_2_ and LDH–COOH exhibited comparatively smaller variations. This behavior can be attributed to the higher sensitivity of smaller particles to intermolecular interactions, while steric hindrance in larger particles reduces ligand accessibility and binding affinity (Figure 4) [57]. Moreover, the smaller size of unmodified LDH results in a higher number of particles per unit mass, thereby increasing the available surface area and the probability of collisions with biomolecules. The amount of adsorbed protein is therefore governed not only by structural parameters such as size, morphology, and crystallinity, but also by the physicochemical properties of the surface, including hydrophilicity, charge, and functionalization [58].

For both unmodified and functionalized LDH, the highest particle-to-protein ratio led to a decrease in hydrodynamic diameter and PDI, suggesting a decreased aggregation due to steric stabilization effects [59]. Protein–particle interactions are primarily driven by non-covalent forces, including van der Waals interactions, hydrogen bonding, electrostatic interactions, and hydrophobic effects. These interactions can be multivalent, leading to the formation of stable complexes, and may also induce conformational changes in the protein structure. Consequently, the dominant interaction mechanism ultimately dictates the macroscopic behavior of the system [60].

Notably, despite the increase in hydrodynamic diameter and the shift in ζ potential values toward near-neutral conditions, the colloidal systems remained stable in suspension. This stability arises from the combined contribution of steric hindrance and non-electrostatic interactions, which effectively reduce particle aggregation even when electrostatic repulsion is diminished.

The observed increase in hydrodynamic diameter can be ascribed to several processes that include protein–protein interactions, adsorption of protein oligomers, and nanoparticle aggregation [61]. LTB has been reported to oligomerize [62]; the presence of such oligomers could influence the adsorption behavior by changing not only the size of the adsorbing molecules, but the interactions with the nanoparticle surface. Furthermore, interactions between adsorbed protein molecules could favor additional protein association, which could potentially contribute to multilayer formation. Nevertheless, since the oligomeric state of rpZDIII was not characterized in the present study, the relative contributions of oligomerization and multilayer adsorption cannot be established from the measured changes in hydrodynamic diameter. In addition, repeated centrifugation-resuspension cycles during washing may promote nanoparticle aggregation and alter the measured colloidal properties [63].

**Figure 5:**
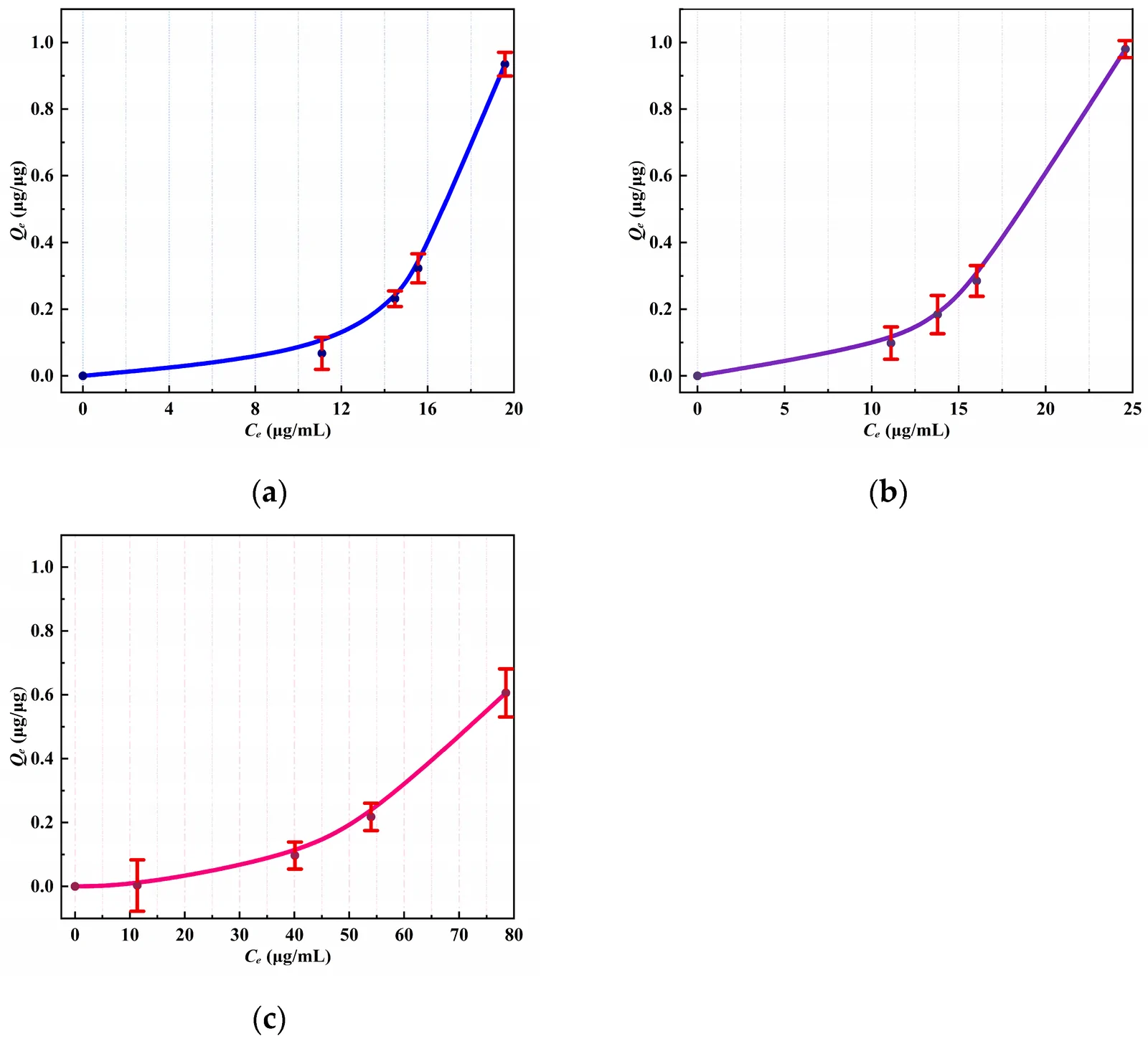
Freundlich adsorption isotherms for the recombinant protein rpZDIII adsorbed on (**a**) LDH, (**b**) LDH–NH_2_, and (**c**) LDH–COOH. The experimental adsorption capacity (*Q_e_*) as a function of equilibrium protein concentration (*C_e_*) was used to fit the Freundlich model. Data are presented as average ± standard deviation (SD) from three independent experiments.

The Freundlich model ($Q_{e}=KC_{e}^{n}$) was fitted to the experimental rpZDIII adsorption isotherms to describe the relationship between equilibrium protein concentration and adsorption capacity on LDH, LDH–NH_2_, and LDH–COOH (Figure 5). The fitted parameters and goodness-of-fit statistics are presented in Table S2. Based on these results, LDH–NH_2_ showed the greatest adsorption capacity over the studied concentration range, followed by LDH and LDH–COOH. The Freundlich exponent (*n*) describes the concentration dependence of adsorption in this empirical model; however, its value does not independently establish a specific adsorption mechanism. Even though the behavior observed could be compatible with surface heterogeneity, protein–protein interactions, or cooperative adsorption [64], these mechanisms cannot be distinguished from the model fit. Moreover, since the oligomeric state of rpZDIII was not characterized, the potential contribution of this state to the adsorption behavior remains to be established.

**Figure 6:**
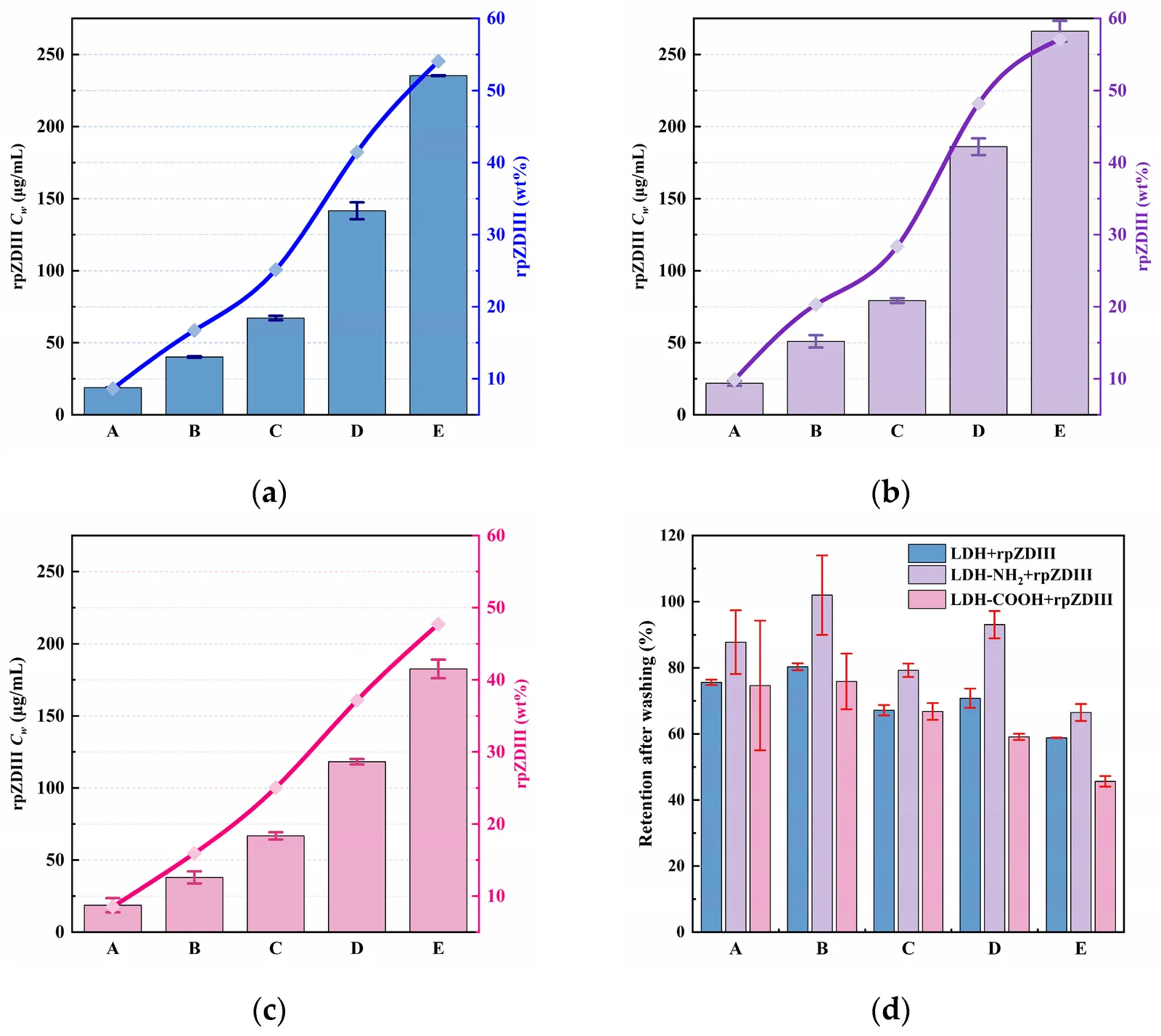
SDS-PAGE protein quantification (*C_w_*) after washing, expressed as remaining protein concentration (bars, left axis) and weight % (lines, right axis) of rpZDIII adsorbed on (**a**) LDH, (**b**) LDH–NH_2_, and (**c**) LDH–COOH at different particle-to-protein ratios. (**d**) Antigen retention after washing. Labels A–E correspond to particle-to-protein ratios of 1:0.125, 1:0.25, 1:0.5, 1:1, and 1:2, respectively. Data are presented as average ± standard deviation (SD) of three independent experiments.

Figure 6 shows the results of rpZDIII that remained adsorbed on the nanoparticles after washing. Protein adsorption was performed at pH 8.1, a condition at which LDH and LDH–NH_2_ had a near-zero net surface charge. Nevertheless, both systems displayed favorable rpZDIII adsorption, highlighting that electrostatic interactions alone cannot fully account for the observed adsorption behavior. LDH–NH_2_ had the highest rpZDIII adsorption across all particle-to-protein ratios, followed by unmodified LDH and LDH–COOH. This trend can be attributed to multivalent interactions and enhanced affinity toward organic molecules [65], particularly considering the predominantly negative surface charge of rpZDIII under the experimental conditions.

### 3.3. FT-IR of Functionalized LDH and Loaded with rpZDIII

**Figure 7:**
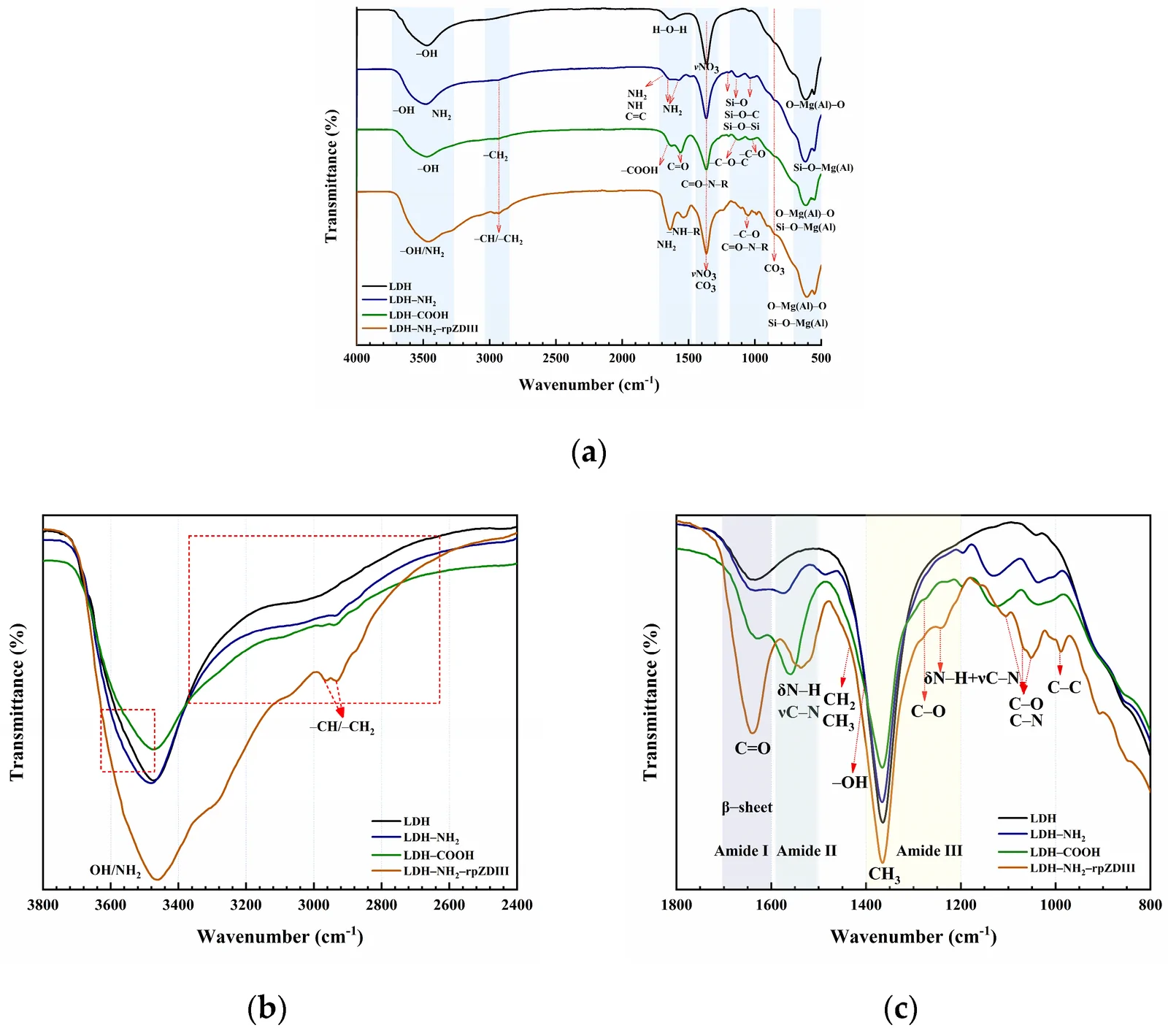
ATR/FT-IR spectra of LDH, LDH–NH_2_, LDH–COOH, and LDH–NH_2_–rpZDIII samples, (**a**) in the 4000–500 cm^−1^ range; (**b**) enlarged view of the 3800–2400 cm^−1^ region showing contributions from –OH/–NH_2_ and C–H/CH_2_ stretching vibrations associated with organic functionalization; and (**c**) enlarged view of the 1800–800 cm^−1^ region, highlighting characteristic bands for C=O, C–N, and C–O, as well as the amide I, amide II, and amide III regions (shaded in purple, green, and yellow, respectively), confirming the presence of protein-related functional groups in the LDH–NH_2_-rpZDIII sample. The spectra correspond to single samples analyzed for each material.

The vibrational modes corresponding to LDH are presented in Figure 7, together with those associated with subsequent functionalization steps and passive adsorption with the recombinant protein.

The broad absorption band centered at 3470 cm^−1^ corresponds to the O–H stretching vibration of hydroxyl groups associated with interlayer water molecules. For LDH, the band at 1640 cm^−1^ corresponds to the H–O–H bending vibration of molecular water. The intense signal in the 1360–1370 cm^−1^ region indicates the presence of interlayer water molecules and nitrate anions, attributed to the asymmetric stretching vibration (ν_3_) of NO_3_^−^ species. Due to the higher affinity of the interlayer environment for multivalent anions (arising from weak electrostatic interactions), this signal may also partially originate from the asymmetric stretching vibration of carbonate anions. A gradual decrease in its intensity is observed as the Mg-Al molar ratio increases [66,67,68]. Additionally, the band in the 850–860 cm^−1^ range is assigned to the weak out-of-plane bending mode (ν_2_) of carbonate [69]. The band at 1648 cm^−1^ corresponds to the bending vibration of water molecules located within the interlayer domain [70]. The peaks in the 620–540 cm^−1^ range correspond to Al–O and Mg–O bond vibrations within the hydroxide layers [71].

Following LDH silanization, the FT-IR spectrum shows the emergence of bands at 1485 and 1574 cm^−1^, assigned to the deformation and scissoring bending modes of the NH_2_ group, respectively. Both bands are characteristic of APTES-based silanization. The band at 1635 cm^−1^ corresponds to the bending mode of H_2_O, as well as to NH, NH_2_, or C=O functional groups [16,72]. The newly formed band at 1200 cm^−1^ is related to silane stretching vibrations, supporting the existence of siloxane bridges [73]. The increase in intensity of the band at 619 cm^−1^ corresponds to deformation vibrations of the Si–O–Mg(Al) bond [74]. Moreover, the LDH–NH_2_ sample exhibits two additional bands at 1130 and 1040 cm^−1^, which correspond to the asymmetric stretching vibrations of Si–O–Si bonds and the symmetric stretching vibrations of Si–O bonds, respectively [75]. The appearance of a weak band at 2932 cm^−1^ corresponds to the asymmetric stretching mode of CH_2_ groups. The NH_2_ vibrational bands of the APTES molecule overlap with the O–H stretching vibrations in the 3300–3500 cm^−1^ region, while the N–H stretching vibration of secondary amines appears near 3290 cm^−1^ [76]. Consequently, a broad band composed of overlapping lower-intensity signals is observed in this region, which can be deconvoluted to resolve individual vibrational modes.

The deconvolution of the 2400–3800 cm^−1^ region reveals a redistribution of O–H-related vibrational contributions upon silanization. Unmodified LDH exhibits bands at 3682 and 3612 cm^−1^, assigned to Al/Mg–OH stretching modes, along with an intense band at 3482 cm^−1^ arising from both structural hydroxyl groups and adsorbed/interlayer water. The band at 3295 cm^−1^ is associated with hydrogen bonding between –OH groups and interlayer water [77], while the broad feature at 3079 cm^−1^ is attributed to interactions between interlayer anions and H_2_O (Figure S5a).

Following silanization, marked changes are observed, including the disappearance of the 3682 cm^−1^ band and a shift in the 3482 cm^−1^ band toward higher wavenumbers, accompanied by a reduction in intensity. This behavior is attributed to the contribution of N–H stretching vibrations introduced by APTES [78]. New bands at 3379 and 3142 cm^−1^ are assigned to primary amine groups, whereas the bands at 2931 and 2921 cm^−1^ correspond to aliphatic –CH_2_ stretching modes (Figure S5b).

Additionally, the bands at 3612 and 3295 cm^−1^ shifted to lower wavenumbers after silanization, consistent with earlier reports that attributed this behavior to the consumption of hydroxyl groups during condensation reactions between LDH and APTES [79]. The pronounced shift in the 3295 cm^−1^ band to 3142 cm^−1^ further indicates overlapping O–H and N–H contributions and enhanced hydrogen-bonding interactions. In this context, stronger hydrogen bonding is known to induce elongation and weakening of the N–H bond, resulting in a red shift in the corresponding vibrational frequency [80]. The band at 3379 cm^−1^ is therefore consistent with N–H stretching of grafted amines, supporting the successful covalent functionalization of LDH with APTES [81].

The appearance of two weak bands at approximately 2978 and 2939 cm^−1^ in the LDH–COOH spectrum is associated with the aliphatic methylene (–CH_2_) groups of the succinamic acid derivative. The shift in these bands toward lower wavenumbers relative to those reported for succinic acid is attributed to amide formation, as well as contributions from hydrogen bonding and conformational changes [82,83]. Moreover, succinic acid exhibits a characteristic broad O–H stretching band in the 3300–2500 cm^−1^ region, which masks the C–H stretching vibrations (Figure 7b). Consequently, although only weak peaks are discernible in the LDH–COOH spectrum, an overall increase in transmittance percentage is observed in comparison with previous samples [84]. Furthermore, an intense band in the 1630–1560 cm^−1^ region is observed (Figure 7c), which is attributed to the asymmetric stretching vibrations of carboxylate groups [85].

The aminated samples (LDH–NH_2_), after subsequent passive adsorption with the recombinant protein rpZDIII (LDH–NH_2_-rpZDIII), exhibit two characteristic bands at 1640 and 1537 cm^−1^, which are assigned to the amide I and amide II bands, respectively. The amide I band arises from the C=O stretching vibration, with minor contributions from C–N stretching and N–H bending vibrations [86]. The amide II band arises from a combination of in-plane N–H bending and C–N stretching vibrations, with weaker contributions from in-plane C=O bending and C–C and N–C stretching [87]. The positions of the amide I and amide II bands indicate an increased content of β-sheet secondary structure in the LDH–NH_2_-rpZDIII sample, which is associated with protein folding (Figure S6). The secondary structure of the recombinant rpZDIII protein was evaluated by deconvolution of the FT-IR spectrum encompassing the amide I, II, and III regions, as well as the 3800–2600 cm^−1^ region, using Gaussian curve fitting. The amide I band is highly sensitive to changes in the protein secondary structure, whereas the amide II band is associated with hydrogen-bonding environments [88]. Deconvolution of the amide I region revealed that rpZDIII is characterized by bands at 1618–1620 cm^−1^ and antiparallel β-sheet contributions at 1682–1708 cm^−1^, together with a minor α-helical component at 1649 cm^−1^ and β-turns at 1671 cm^−1^ [89] (Figure S7). This predominance of β-sheet motifs is consistent with the previously reported AlphaFold structural prediction [90].

Following adsorption onto LDH–NH_2_, changes in the relative contribution of the amide I components suggest conformational rearrangements associated with protein–surface interactions. In particular, a decrease in α-helical contributions and an increase in β-sheet-associated bands were observed, although partial masking of α-helical vibrations by overlapping β-sheet and water bands cannot be excluded [91,92].

As shown in Figure S6a, the deconvoluted bands at 1683 and 1640 cm^−1^ indicate that the secondary structure is predominantly composed of β-sheet motifs, with a lower contribution from β-turns. The low contribution of α-helical structures may result from overlap with H_2_O and interlayer H–O–H bending vibrations that partially mask the amide I region [92].

Additional bands at 1438, 1394, 1362, and 1322 cm^−1^ were assigned to protein side-chain vibrations and overlapping contributions from NO_3_^−^/CO_3_^2−^ species present in the LDH structure [93]. The deconvoluted bands at 1640, 1547, and 1394 cm^−1^ in the LDH–NH_2_-rpZDIII sample are consistent with amide I (C=O stretching), amide II (N–H bending), and COO^−^ vibrations from amino acid side chains, respectively, in agreement with FT-IR spectra previously reported for Zika virus proteins [94]. Additionally, the band observed at 1105 cm^−1^ may be assigned to C–O and C–N stretching vibrations of protein side chains, including residues such as serine, threonine, and histidine [95,96], which are well represented in the rpZDIII sequence (Figure 7c) [90].

Deconvolution of the 3800–2600 cm^−1^ region revealed bands at approximately 3300 and 3050 cm^−1^, assigned to amide A and amide B vibrations, respectively (Figure S6b) [97]. Their partial overlap with the broad O–H stretching band reflects the high water content of the system [98]. The increased intensity observed in this region relative to unloaded LDH provides additional evidence of successful rpZDIII adsorption onto LDH–NH_2_.

### 3.4. TGA of Functionalized LDH and Loaded with rpZDIII

**Figure 8:**
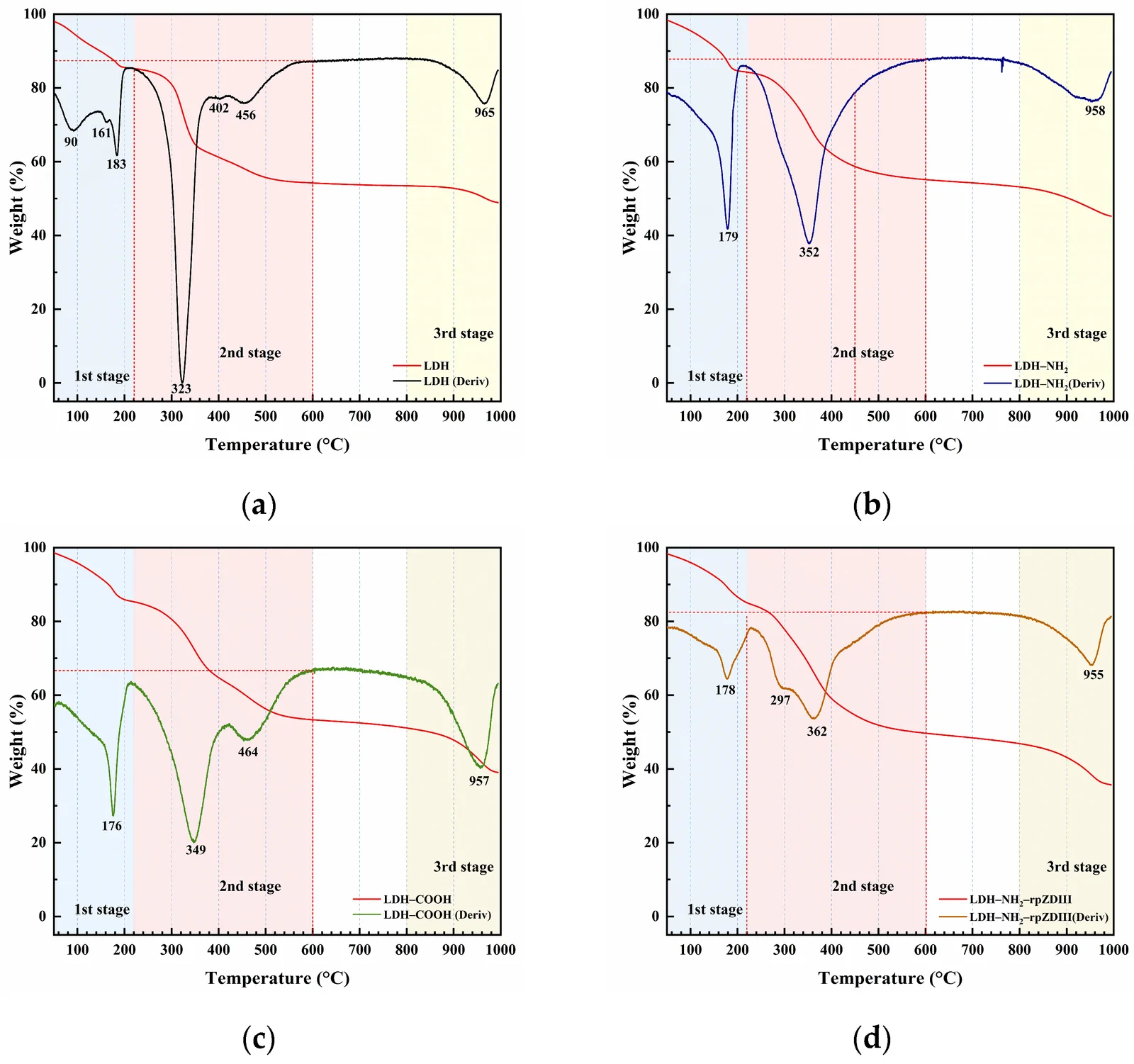
TGA and DTG profiles of (**a**) LDH, (**b**) LDH–NH_2_, (**c**) LDH–COOH, and (**d**) LDH–NH_2_-rpZDIII. Three main stages are identified: first, dehydration (<220 °C, blue region); second, dehydroxylation and organic decomposition (220–600 °C, red region); and third, decomposition of residual inorganic species (>800 °C, yellow region). A characteristic peak at ~323 °C is observed for all samples, while additional features at ~340–375 °C and ~460 °C emerge upon functionalization, consistent with the incorporation of silane and biomolecular species. The TGA and DTG profiles correspond to single samples analyzed for each material.

The thermogravimetric (TGA) and derivative thermogravimetric (DTG) profiles are shown in Figure 8 for the materials studied. In general, mass losses in the 30–120 °C range are associated with the removal of physisorbed water molecules from the material, while the weight loss observed between 120 and 170 °C corresponds to interlayer water elimination from the LDH structure [99]. This dehydration process is observed in all samples, with the main event occurring between 175 and 180 °C. The mass losses observed at approximately 950 °C are attributed to the decomposition of sulfate, carbonate, and nitrate species, as well as to the formation of metal oxides, phase transformation, and structural reorganization of the material [100,101].

The original, unmodified LDH sample exhibits multiple dehydration steps (DTG peaks at 90, 161, and 184 °C), which can be attributed to different water interaction modes, including surface adsorption, interlayer confinement, and hydrogen bonding. The main dehydration event at 184 °C shifted relative to the other samples, indicating increased thermal stability in the presence of hydrogen-bonding interactions [102]. The mass loss observed in the 170–280 °C range can be attributed to overlapping processes, including dehydroxylation and the partial decomposition of interlayer carbonate anions associated with Al–OH groups. These processes occur simultaneously with the dehydroxylation of Mg–OH groups, which becomes more prominent at approximately 323 °C [99,103] (Figure 8a).

For the LDH–NH_2_ sample, an intense peak at 179 °C is observed, which is associated with water loss and the release of physisorbed, polycondensed silane species. This behavior can be explained by the lower thermal stability of APTES molecules bound through hydrogen bonding compared to those that are covalently attached, which decompose at higher temperatures [104,105] (Figure 8b). Analyzing chemisorbed silane species, the mass loss observed in the 300–500 °C range is attributed to the decomposition of alkyl chains from organic moieties, the cleavage of C–Si bonds, and the breakdown of grafted silane structures. The broadness of the DTG band in this region suggests multiple overlapping thermal degradation steps associated with different APTES binding modes. These include silane species grafted at both the edges and basal surface of the material, variations in the number of chemical bonds between APTES and the LDH layers, and differences in surface organization, such as monolayer or multilayer arrangements [106,107].

Deconvolution of the DTG peaks corresponding to the second stage of thermal degradation reveals a peak at approximately 320–326 °C in all samples, attributed to dehydroxylation. Additionally, peaks in the 340–375 °C range emerge after functionalization and antigen-loading of the material, indicating the contribution of organic moieties (Figure S8b).

The LDH–COOH sample exhibits DTG peaks at 326, 351, and 464 °C. The first peak is associated with the dehydroxylation of Mg–OH groups, whereas the peaks at 351 and 464 °C correspond to the two-step degradation of the organic functionality. This process begins with the decomposition of the silane aliphatic chains and the cleavage of Si–C bonds, which subsequently propagates toward the carboxyl group [108,109] (Figure S8c).

For the LDH–NH_2_–rpZDIII sample (prepared using a particle-to-antigen ratio of 1:0.125), changes in the DTG band shape during the second stage of thermal decomposition, along with a slight shift toward higher temperatures, suggest possible interactions between rpZDIII and the LDH–NH_2_ surface that may influence the thermal degradation behavior of the nanovaccine [110]. Since proteins undergo complex, multistep thermal decomposition rather than a single, well-defined event, researchers have reported their degradation over broad temperature ranges, such as 270–493 °C [111] and 200–450 °C. DTG peak deconvolution revealed contributions previously associated with LDH dehydroxylation (323 °C) and silane decomposition (343 °C), along with three additional features at approximately 295, 370, and 428 °C. These additional signals may reflect protein-related thermal degradation processes, including structural rearrangements and decomposition of organic components [112]. In particular, the feature at 295 °C could relate to the thermal decomposition of amino acid residues, given that histidine has been reported to undergo thermal degradation at approximately 280 °C [113]. Similarly, the features at 370 and 428 °C occur within temperature regions previously associated with peptide bond cleavage and the decomposition of other chemical linkages in protein-based materials [114]. Nevertheless, these assignments should be considered tentative, as the overlapping thermal contributions from rpZDIII, the LDH matrix, and surface functional groups prevent the unequivocal identification of individual molecular degradation processes based on TGA/DTG data alone. Furthermore, the shift toward higher temperatures may suggest changes in the thermal stability of the adsorbed protein due to interactions with the LDH-NH_2_ surface, which could act as a stabilizing matrix or physical barrier against thermal degradation. Similar behavior has been reported for other protein-inorganic support systems [110,111]. Therefore, the changes observed in the DTG profile are consistent with protein incorporation and possible protein–surface interactions. Additional analytical techniques would be required to confirm the specific molecular degradation mechanisms (Figure S8d).

To further quantify the contribution of each functionalization step to the overall thermal behavior, the mass loss associated with specific temperature ranges was analyzed. For the silanization of LDH (LDH–NH_2_), the mass loss was evaluated over the temperature range 120–450 °C, corresponding to the first and second stages of organic decomposition, yielding a total mass loss of 1.48%. A 3.68% difference in total mass loss between LDH and LDH–NH_2_ at the end of the thermogravimetric analysis was also determined. These values are comparable to the quantification of primary amines by the ninhydrin assay (2.67%). The efficiency of APTES functionalization can be estimated by considering a theoretical concentration of grafted primary amines after silanization, which was estimated as 3.41% (820 µM). Therefore, the amination efficiency of LDH corresponds to a functionalization range of 43.5–78.2%. The discrepancy between the theoretical and experimentally determined values may be attributed to limitations in grafting density, governed by the availability of surface hydroxyl groups and steric hindrance during condensation reactions. In addition, excessive silane crosslinking may restrict interlayer accessibility or reduce the effective surface area, thereby reducing organic loading [115].

For the SA-functionalized sample (LDH–COOH), the temperature range of 220–550 °C was selected since it provides a more selective estimate of the mass loss attributable to the incorporation of –COOH groups. This selection is supported by the appearance of a prominent DTG band not observed in the other samples, which is associated with the carboxylation reaction. The mass loss within this temperature window (2.85%) agrees well with the value calculated from the ninhydrin assay (2.85%), validating this approach.

For rpZDIII adsorption, although the temperature range of 220–600 °C encompasses the main degradation events associated with the biomolecule, it does not capture its full contribution. The adsorbed protein may exhibit enhanced thermal stability due to surface interactions with LDH–NH_2_, shifting its degradation toward higher temperatures. Therefore, the total mass loss (13.22%) at the end of the TGA analysis is considered more representative of the protein’s overall contribution relative to unmodified LDH, as shown in Figure 8. These results provide indirect evidence of protein incorporation onto the LDH–NH_2_ matrix.

### 3.5. Immunogenicity Assessment

Given that differences in adsorption existed when loading rpZDIII onto LDH, LDH–NH_2_, and LDH–COOH, adjusting the antigen concentration to 25 μg/mL after washing resulted in different nanoparticle concentrations in the final immunization formulations. The initial particle-to-antigen mass ratio during adsorption was 1:2. Antigen retention was calculated as the percentage of protein remaining after washing relative to the concentration adsorbed before washing, which generated values of 71, 86, and 64% for LDH, LDH–NH_2_, and LDH–COOH, respectively (Table 2). The nanoparticle concentrations in the final formulations were calculated from the dilution factors and the initial particle concentration of 200 μg/mL, which resulted in concentrations of 25 μg/mL for LDH and LDH–NH_2_ and 36 μg/mL for LDH–COOH. Table 2 summarizes the physicochemical properties of the rpZDIII-loaded LDH formulations after washing, including hydrodynamic size, polydispersity index (PDI), ζ potential, final antigen and particle masses and concentrations, and antigen retention.

**Figure 9:**
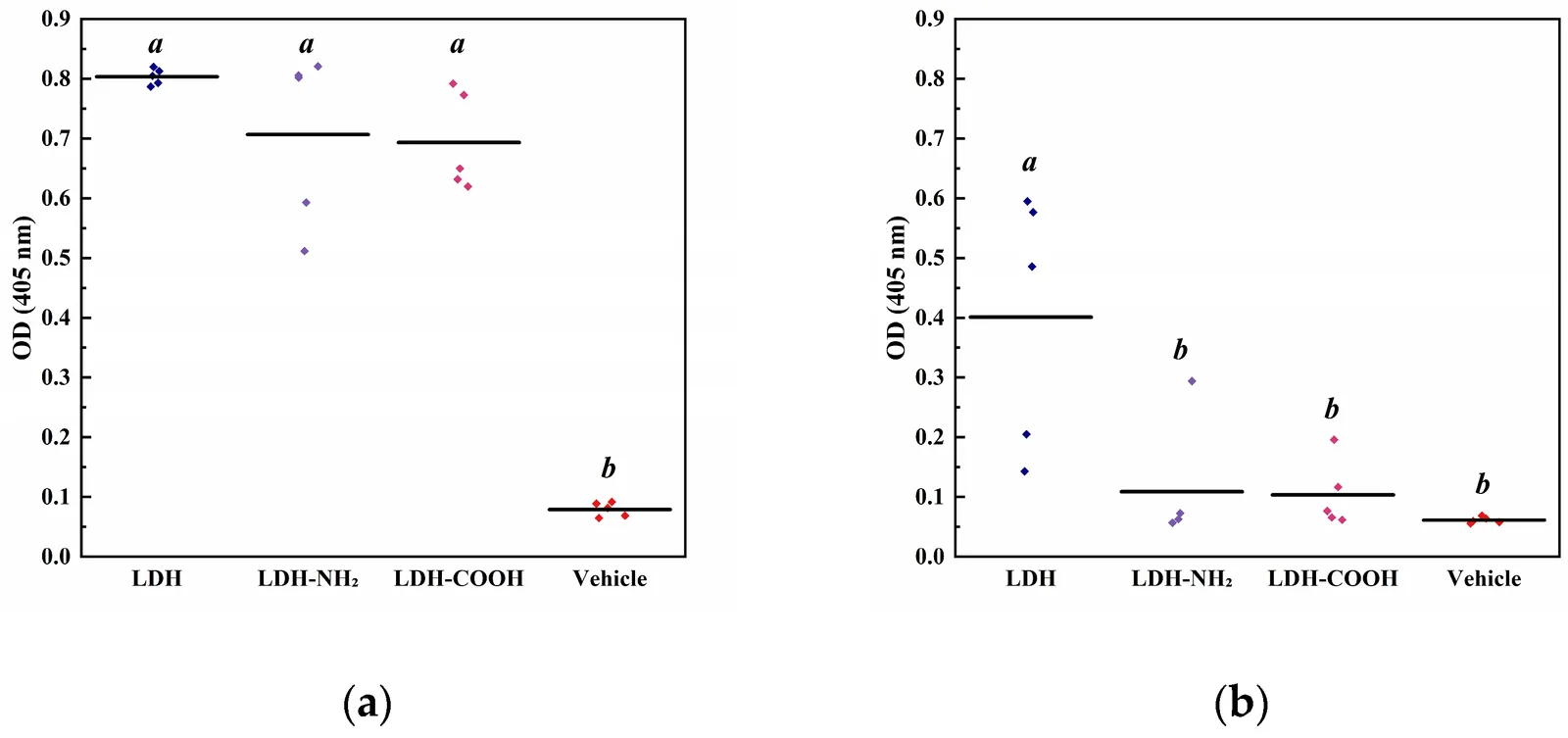
ELISA-determined antibody levels, expressed as optical density (OD) at 405 nm on day 42 (14 days after the last immunization), in sera from animals immunized with LDH, LDH–NH_2_, and LDH–COOH loaded with rpZDIII. Antibody recognition was evaluated against: (**a**) recombinant rpZDIII protein or (**b**) ZIKV E protein. Each serum sample was analyzed once. Individual points represent individual animals (*n* = 5 per group), and horizontal bars indicate the group average. Different letters indicate statistically significant differences among groups according to Tukey’s multiple comparison test following one-way ANOVA (*p* < 0.05).

The ELISA results (Figure 9) showed that all nanoparticle formulations induced significant antibody responses against rpZDIII. The Tukey’s multiple comparison test showed no statistically significant differences between LDH, LDH–NH_2_, and LDH–COOH, while these three formulations elicited significantly higher responses than the vehicle control. In contrast, antibody recognition of the full-length ZIKV E protein differed among formulations. LDH elicited significantly higher responses than LDH–NH_2_, LDH–COOH, and the vehicle control. No statistically significant differences were detected between these three groups.

A limitation of this study is that the antibody responses were evaluated at a single serum dilution for each target antigen. Therefore, these results reflect relative antibody binding under the assay conditions, and do not establish endpoint antibody titers. In addition, ELISA only measures antigen binding instead of virus neutralization. Therefore, the reported antibody responses cannot be interpreted as evidence of neutralizing activity against ZIKV. Further studies are required to provide evidence of antibody titers and functional activity.

The reduced anti-E protein antibody response observed for LDH–NH_2_ and LDH–COOH, despite LDH-NH_2_ showing higher rpZDIII retention, suggests that antigen loading by itself does not determine recognition of the target protein. One potential explanation is that the surface chemistry influences the orientation or conformation of the adsorbed antigen, affecting the accessibility of ZIKV E-derived epitopes. Interactions between rpZDIII and the functionalized surfaces could partially mask these epitopes, reducing their ability for recognition even when a greater antigen concentration remains adsorbed to the nanoparticles. Nevertheless, antigen orientation and conformational changes were not characterized in this study; therefore, these considerations remain hypothetical. Another consideration is that rpZDIII is composed of a ZIKV envelope domain III-derived antigen fused to the B subunit of the heat-labile enterotoxin (LTB). Accordingly, the antibody response measured against rpZDIII may include antibodies directed against the derivative antigen from ZIKV or the LTB moiety, while the full-length ZIKV E protein used does not contain LTB. Therefore, the strong antibody responses against rpZDIII recorded for all formulations do not necessarily imply equivalent recognition of the epitopes present in the full-length ZIKV E protein. Even though the present results do not establish the relative contribution of these mechanisms, they suggest that the nanoparticle surface chemistry can affect both antigen loading and retention, and accessibility and recognition of ZIKV-derived epitopes. Additional studies will be required to distinguish the contribution of these possibilities.

An additional consideration is that, despite having a standardized dose of 5 μg per immunization, the nanoparticle mass administered was not fixed because of differences in antigen loading. Therefore, the contribution of nanoparticle dose to the observed immune responses cannot be completely separated from the effects of surface chemistry and antigen–particle interactions. Differences in the amount of carrier administered could have influenced the adjuvant effect of the formulations; nevertheless, this contribution was not independently studied. In addition, a soluble rpZDIII control was not included in the immunization study. Therefore, the results presented in this study do not establish if the studied nanovaccines elicit stronger antibody responses than the soluble antigen. These limitations must be considered when analyzing the differences in recognition observed in the formulations studied. Future studies should evaluate the effect of both antigen and nanoparticle dose.

The reduced anti-E protein antibody response observed for LDH–NH_2_ and LDH–COOH could indicate that surface modification altered the adsorption behavior of the recombinant rpZDIII protein. Changes in protein orientation or conformation upon adsorption could have reduced the exposure of ZIKV E epitopes while preserving accessibility of LTB-associated epitopes. However, additional characterization would be required to confirm this hypothesis.

## 4. Conclusions

This study demonstrated that the surface chemistry of LDH nanoparticles can be modified through silanization and subsequent carboxylation, resulting in significant changes in particle size, surface charge, point of zero charge, protein adsorption behavior, and biological performance. The successful incorporation of amino and carboxyl functionalities was confirmed by ζ potential measurements, ninhydrin assays, FT-IR spectroscopy, and thermogravimetric analysis.

Adsorption studies using BSA and α-CHY as model proteins revealed that surface functionalization strongly influences both adsorption capacity and adsorption stability. Moreover, washing experiments demonstrated that adsorption capacity alone is insufficient to predict protein retention, highlighting the importance of evaluating adsorption stability when designing nanoparticle-based delivery systems.

The recombinant rpZDIII antigen was successfully adsorbed onto all nanoparticle formulations, and its adsorption behavior was consistent with heterogeneous surface interactions described by the Freundlich model. LDH–NH_2_ exhibited the highest antigen adsorption and retention after washing, while FT-IR and thermogravimetric analyses confirmed successful protein immobilization and increased thermal stability of the adsorbed antigen.

All nanoparticle formulations maintained high HEK-293T cell viability at the concentrations used for immunization, supporting their preliminary in vitro cellular compatibility. Nevertheless, these results do not establish the overall safety of the nanovaccines. Further experimentation is required in this regard, including in vivo studies. Immunogenicity studies demonstrated that the three LDH-based formulations elicited robust antibody responses against the recombinant rpZDIII protein. However, only the unmodified LDH induced antibodies that significantly recognized the full-length ZIKV envelope protein relative to the control group. These findings suggest that enhanced antigen loading does not necessarily translate into improved immunological recognition of relevant viral epitopes and that nanoparticle surface functionalization may influence antigen orientation, conformation, or epitope accessibility following adsorption.

Overall, the results demonstrate that the LDH surface chemistry has a key role in regulating protein adsorption, antigen retention, and immune recognition. The study highlights the importance of balancing antigen loading with preservation of antigenic determinants during nanovaccine design and identifies unmodified LDH as the most effective formulation for maintaining the immunogenicity of the ZIKV envelope epitopes evaluated in this work.

## Figures and Tables

**Table 1:** Particle-to-protein mass ratios evaluated during the passive adsorption onto LDH nanoparticles at a fixed concentration (200 µg/mL) with BSA or α-CHY in increasing concentrations (starting from 25 to 400 µg/mL).

Label	Mass Ratio (LDH:Protein)	Particle Conc. (μg/mL)	Protein Conc. (μg/mL)
A	1:0.125	200	25
B	1:0.25	200	50
C	1:0.5	200	100
D	1:1	200	200
E	1:2	200	400

**Table 2:** Physicochemical properties of the rpZDIII-loaded LDH formulations after washing.

Nanoparticle	Size (nm)	PDI	ζ Potential (mV)	Antigen Conc. (μg/mL)/Antigen per Dose (μg)	Particle Conc. (μg/mL)/Particle Mass per Dose (μg)	Antigen Retention (%)
LDH	197	0.09	−20	25/5	25/5	71
LDH–NH_2_	161	0.14	−22	25/5	25/5	86
LDH–COOH	147	0.10	−19	25/5	36/7	64

Values represent single measurements from formulations prepared in one experiment; no standard deviations are reported. Final nanoparticle concentrations were calculated from the initial nanoparticle concentration and the dilution factors applied after washing, assuming no nanoparticle loss.

## Data Availability

The data supporting the results of this study are available in the article and its Supplementary Materials. Additional inquiries can be directed to the corresponding authors.

## Supplementary Materials

The following supporting information can be downloaded at https://www.mdpi.com/article/10.3390/nano16211419/s1, Figure S1: Size distributions of Mg-Al layered double hydroxide (LDH) nanoparticles after their synthesis and functionalization; Figure S2: HRTEM images and corresponding FFT/IFFT analyses of Mg–Al LDH nanoparticles; Figure S3: Adsorption behavior of model proteins on pristine and surface-functionalized LDH particles; Figure S4: Hydrodynamic size and ζ potential of pristine and surface-functionalized LDH particles after interaction with model proteins at increasing particle-to-protein ratios; Figure S5: Deconvolution of the FTIR spectra (2400–3800 cm^−1^) of (a) LDH and (b) LDH–NH_2_; Figure S6: Deconvoluted FT-IR spectrum of the LDH–NH_2_–rpZDIII sample obtained using Gaussian curve fitting; Figure S7: Deconvolution of the amide I region of rpZDIII protein showing dominant β-sheet contributions and minor α-helix and β-turn components; Figure S8: DTG peak deconvolution of (a) LDH, (b) LDH–NH_2_, (c) LDH–COOH, and (d) LDH–NH_2_–rpZDIII; Table S1: Langmuir isotherm parameters for the adsorption of model proteins (BSA and α-CHY) on pristine and surface-functionalized LDH systems; Table S2: Freundlich isotherm parameters for the adsorption of rpZDIII on pristine and surface-functionalized LDH systems; Table S3: Physicochemical characterization of pristine LDH and rpZDIII-loaded LDH systems at different particle-to-protein ratios; Table S4: Physicochemical characterization of amine-functionalized LDH and rpZDIII-loaded LDH–NH_2_ systems at different particle-to-protein ratios; Table S5: Physicochemical characterization of carboxyl-functionalized LDH and rpZDIII-loaded LDH–COOH systems at different particle-to-protein; Section: Calculating the amount of reactant to functionalize nanoparticles in suspension. References [107,116,117,118,119] are cited in the supplementary materials.
