# Supplementary material for: Surface Chemistry Engineering of Layered Double Hydroxides for Antigen Adsorption in Zika Virus Nanovaccine Development
Source: Nanomaterials (Basel). 2026 Nov 4;16(21):1419. doi: 10.3390/nano16211419 (PMC13764742; doi:10.3390/nano16211419)
Supplement: Supplementary file 1 [file nanomaterials-16-01419-s001.zip › nanomaterials-4636514-supplementary.pdf]

Supporting Information

Surface Chemistry Engineering of Layered Double Hydroxides for Antigen Adsorption in Zika Virus Nanovaccine Development

Sayma A. Rodriguez-Montelongo [1], Arleth Miranda-Lopez [1,2], Mariana Sanchez-Gil [1,2], Lourdes Betancourt-Mendiola [1,2], Veronica Perez-Luna [3], Mauricio Comas-Garcia [3,4], Mariano J. Garcia-Soto [1], Sergio Rosales-Mendoza [1,2,5,*] and Omar Gonzalez-Ortega [1,2,*]

1 Facultad de Ciencias Químicas, Universidad Autónoma de San Luis Potosí, Av. Manuel Nava 6, San Luis Potosi 78210, Mexico; say.mont07@gmail.com (S.A.R.-M.); arlethmiranda@hotmail.com (A.M.-L.); a237003@alumnos.uaslp.mx (M.S.-G.); lourdes.betancourt@uaslp.mx (L.B.-M.); mariano.soto@uaslp.mx (M.J.G.-S.)

2 Sección de Biotecnología, Centro de Investigación en Ciencias de la Salud y Biomedicina, Universidad Autónoma de San Luis Potosí, Av. Sierra Leona 550, San Luis Potosi 78210, Mexico

3 Sección de Microscopía de Alta Resolución, Centro de Investigación en Ciencias de la Salud y Biomedicina, Universidad Autónoma de San Luis Potosí, Av. Sierra Leona 550, San Luis Potosi 78210, Mexico; veronica.perez@uaslp.mx (V.P.-L.); mauricio.comas@uaslp.mx (M.C.-G.)

4 Facultad de Ciencias, Universidad Autónoma de San Luis Potosí, Av. Parque Chapultepec 1570, San Luis Potosi 78210, Mexico

5 Department of Pharmacognosy and Pharmaceutical Botany, Faculty of Pharmaceutical Sciences, Chulalongkorn University, Bangkok 10330, Thailand

* Correspondence: rosales.s@uaslp.mx (S.R.-M.); omar.gonzalez@uaslp.mx (O.G.-O.)

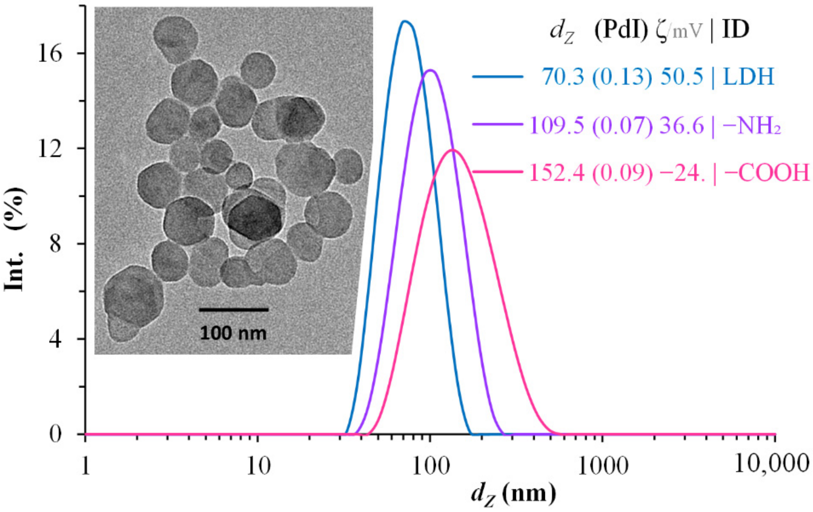

Figure S1. Size distributions of Mg-Al layered double hydroxide (LDH) nanoparticles after their synthesis and functionalization. The slurry obtained by coprecipitating a 3:1 molar ratio of Mg^{2+}:Al^{3+} and then refined via hydrothermal treatment was washed by centrifuging and resuspending in water, rendering monodisperse LDH. **Insert:** TEM image of an LDH suspension. The silanization of LDH with (3-aminopropyl)triethoxysilane (APTES) rendered LDH–NH_2, which were further reacted with succinic anhydride (SA) to obtain LDH–COOH. The hydrodynamic size, polydispersity index (PDI), and ζ potential of LDH, LDH–NH_2, and LDH–COOH are reported in Tables S3, S4, and S5, respectively, with the corresponding standard deviations.

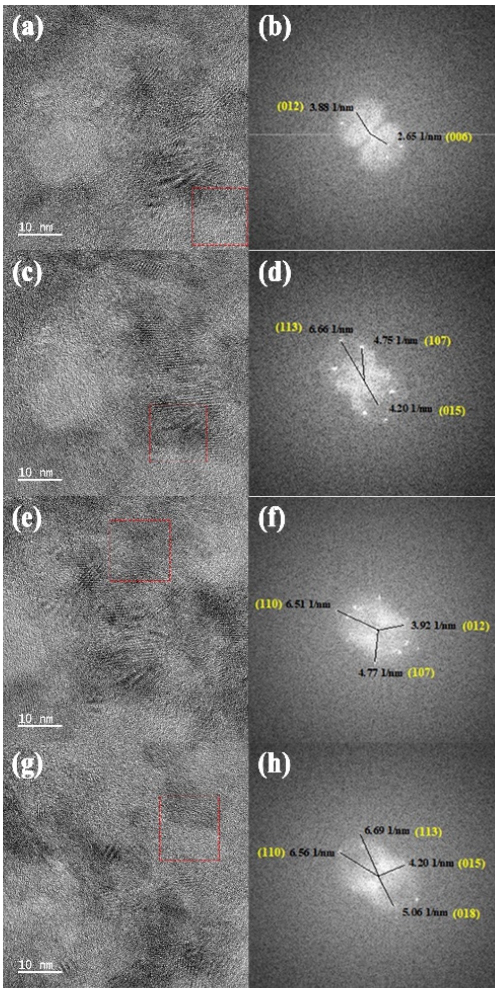

Figure S2. Mg-Al LDH nanoparticles were analyzed by fast Fourier transform (FFT) of selected HR-TEM regions using Gatan DigitalMicrograph. The resulting FFT patterns exhibited well-defined diffraction spots, confirming the presence of local crystalline domains. The corresponding lattice periodicities were further evaluated by inverse fast Fourier transform (IFFT) analysis. The reciprocal-space distances obtained from the FFT patterns and the corresponding interplanar spacings were consistent with characteristic reflections of the Mg-Al LDH structure. The crystallographic planes were assigned based on comparison of the experimentally determined lattice spacings with those reported for Mg-Al LDH structures in the literature [116–119]. A reciprocal-space distance of 2.65 nm^{-1} (d = 0.377 nm) was assigned to the (006) plane, while values of 3.88–3.92 nm^{-1} (d = 0.258–0.255 nm) were assigned to the (012) plane. Reflections at 4.20 nm^{-1} (d = 0.238 nm) and 4.75–4.77 nm^{-1} (d = 0.211–0.210 nm) were indexed to the (015) and (107) planes, respectively. The (018) plane was associated with a reciprocal space distance of 5.06 nm^{-1} (d = 0.198 nm), whereas the (110) and (113) planes were assigned to values of 6.51–6.56 nm^{-1} (d = 0.154–0.152 nm) and 6.66–6.69 nm^{-1} (d = 0.150–0.149 nm), respectively. Variations in diffraction-pattern orientation across the analyzed regions further indicate nanoscale crystalline domains with distinct crystallographic orientations.

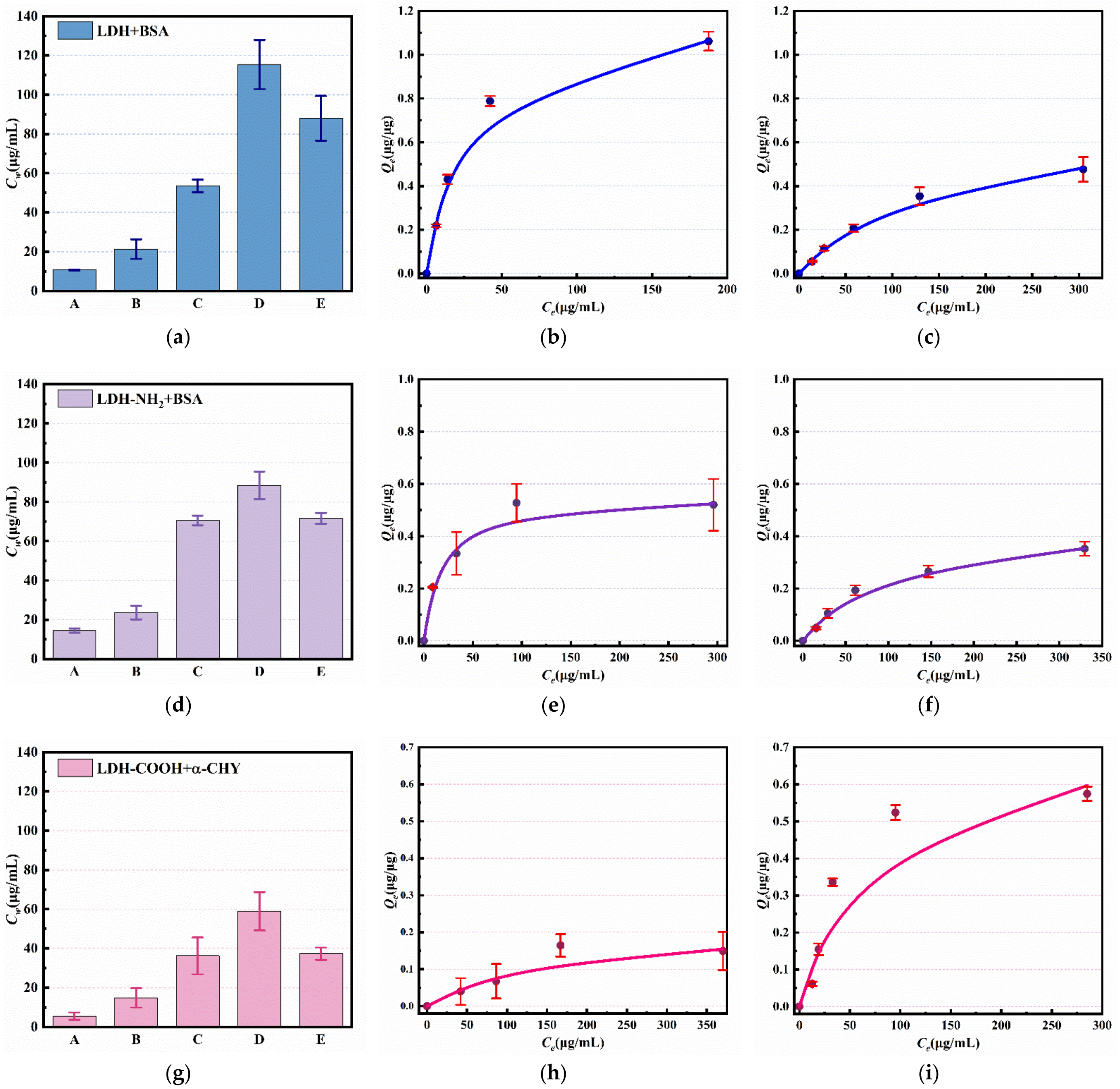

Figure S3. Adsorption behavior of model proteins on pristine and surface-functionalized LDH particles. **(a, d, g)** Protein concentrations quantified by SDS–PAGE after adsorption onto LDH, LDH–NH_2, and LDH–COOH (C_w), respectively, at increasing particle-to-protein ratios (A–E). **(b, e, h)** Adsorption isotherms of BSA on LDH, LDH–NH_2, and LDH–COOH. **(c, f, i)** Adsorption isotherms of α-chymotrypsin (α-CHY) on LDH, LDH–NH_2, and LDH–COOH. Data are presented as average ± standard deviation (SD, n = 3). A–E correspond to particle-to-protein ratios of 1:0.125, 1:0.25, 1:0.5, 1:1, and 1:2, respectively.

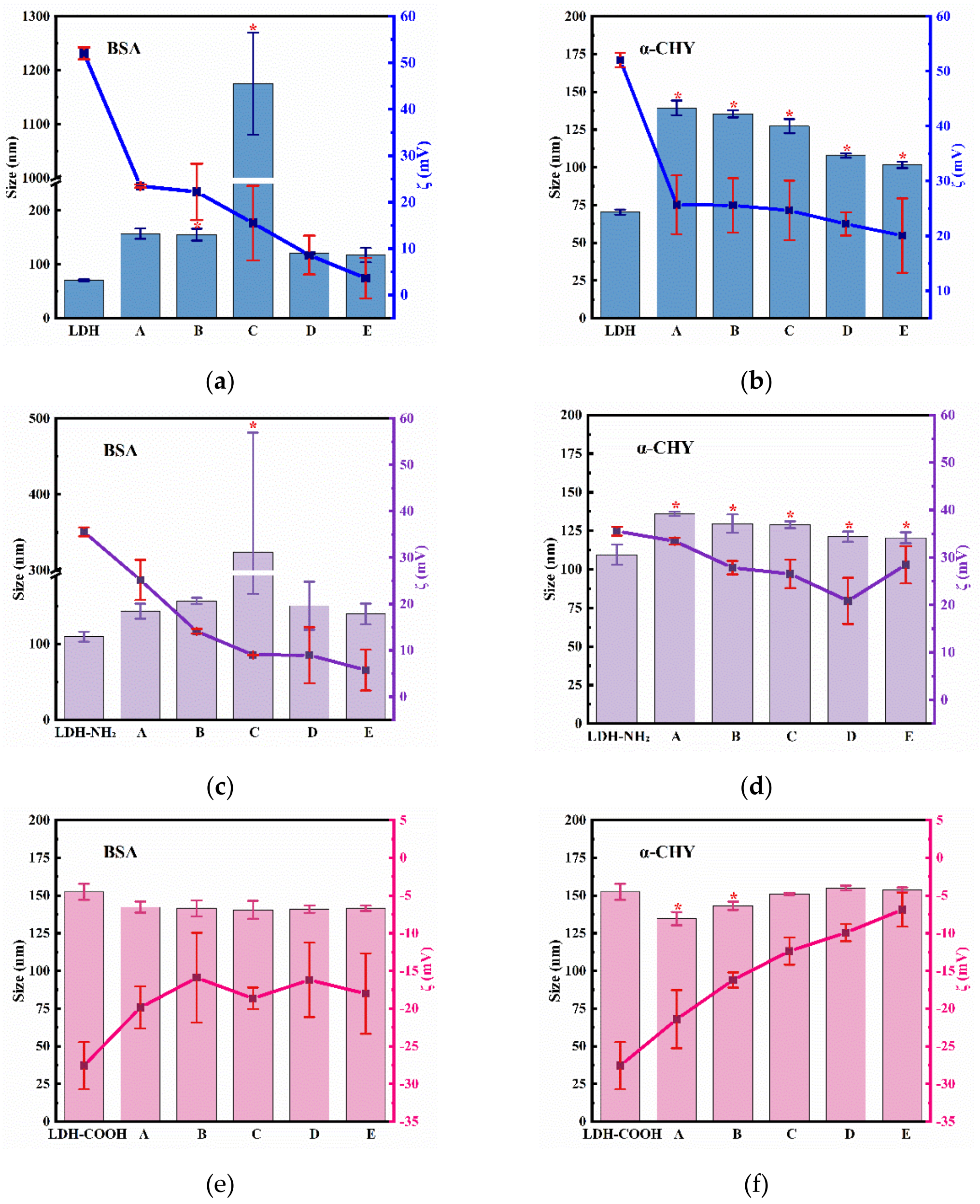

Figure S4. Hydrodynamic size and ζ potential of pristine and surface-functionalized LDH particles after interaction with model proteins at increasing particle-to-protein ratios (A–E). **(a, c, e)** BSA adsorption onto LDH, LDH–NH_2, and LDH–COOH, respectively. **(b, d, f)** *α*-CHY adsorption onto LDH, LDH–NH_2, and LDH–COOH, respectively. Bars represent hydrodynamic size and lines represent ζ potential. Error bars represent standard deviation (SD, n = 3). A–E correspond to particle-to-protein ratios of 1:0.125, 1:0.25, 1:0.5, 1:1, and 1:2, respectively. Statistical significance in hydrodynamic size was evaluated by ANOVA followed by Dunnett's multiple-comparisons test, comparing each protein-treated group with its corresponding control nanoparticles (LDH, LDH–NH_2, or LDH–COOH). Asterisks represent statistically significant differences ($p < 0.05$).

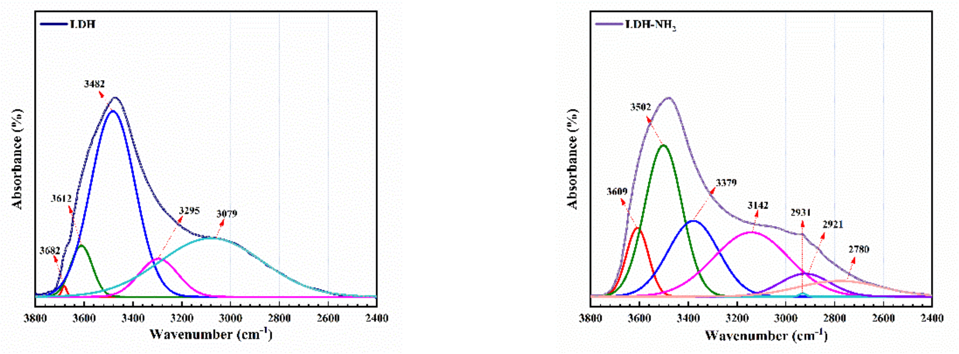

(**a**) (**b**)

Figure S5. Deconvolution of the FTIR spectra (2400–3800 cm^{-1}) of **(a)** LDH and **(b)** LDH-NH_2. LDH shows O–H stretching bands at 3682 and 3612 cm^{-1} and a broad band at 3482 cm^{-1} from hydroxyl groups and adsorbed water. After silanization, the O–H envelope shifts (~3502 cm^{-1}) and new bands at 3379 and 3142 cm^{-1} (N–H) and 2931–2921 cm^{-1} (C–H) appear, confirming surface functionalization with APTES. The spectra correspond to individual measurements.

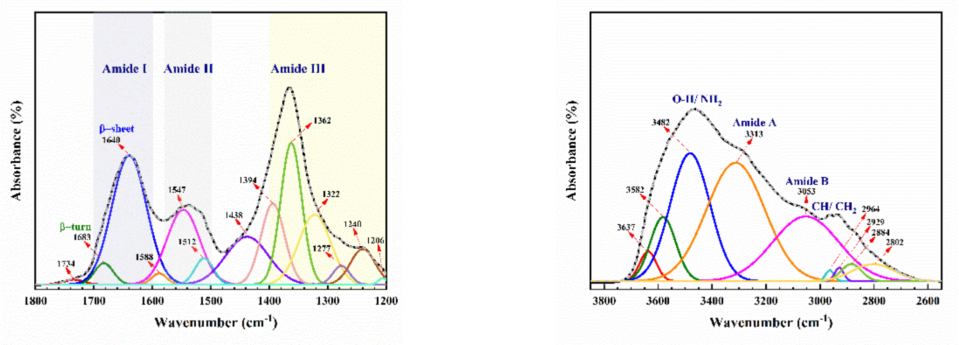

(**a**) (**b**)

Figure S6. Deconvoluted FT-IR spectrum of the LDH–NH_2–rpZDIII sample obtained using Gaussian curve fitting. **(a)** shows the 1800–1200 cm^{-1} region, where the individual components of the amide I, amide II, and amide III bands are resolved, including contributions associated with β-sheet and β-turn secondary protein structures. **(b)** presents the 3800–2600 cm^{-1} region, highlighting the bands corresponding to amide A and amide B, as well as contributions from O–H/NH_2 and C–H/CH_2 stretching vibrations. The spectra correspond to individual measurements.

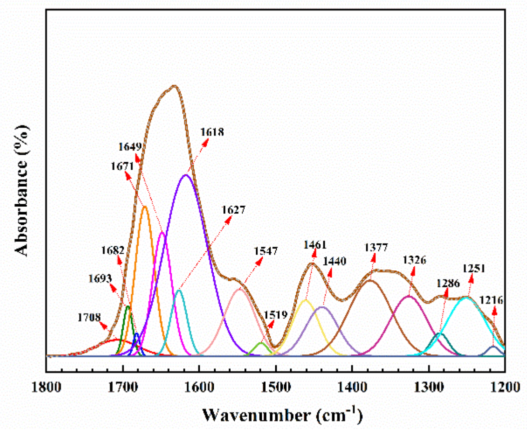

Figure S7. Deconvolution of the amide I region of rpZDIII protein showing dominant β-sheet contributions and minor α-helix and β-turn components. The spectrum corresponds to an individual measurement.

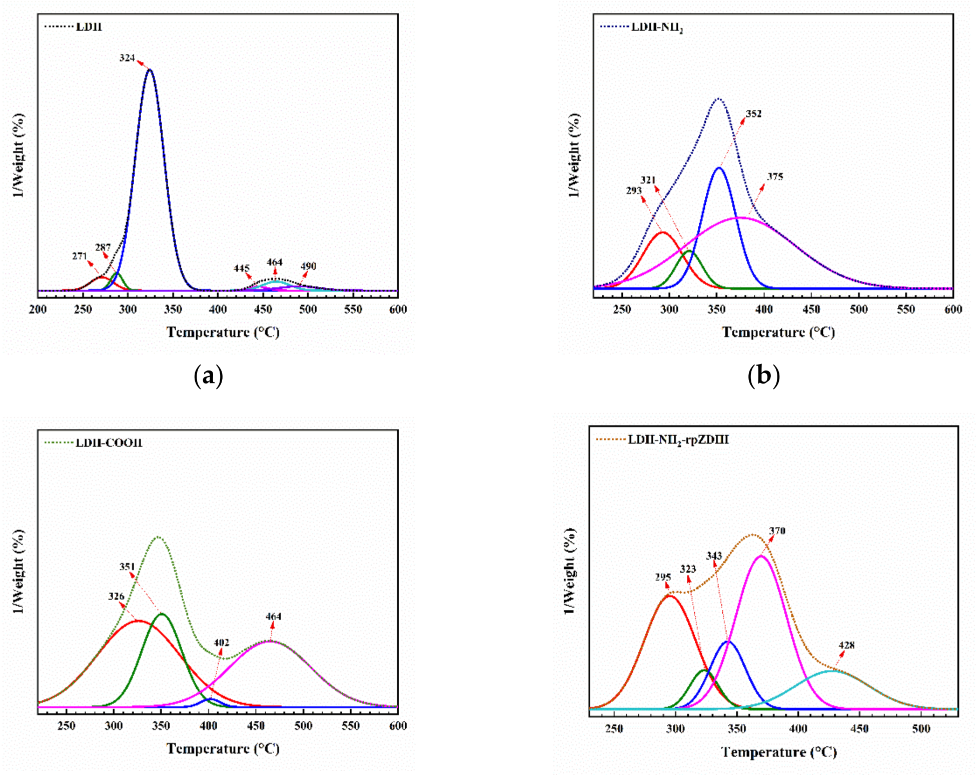

Figure S8. DTG peak deconvolution of **(a)** LDH, **(b)** LDH–NH_2, **(c)** LDH–COOH, and **(d)** LDH–NH_2–rpZDIII, focused on the second stage of thermal decomposition. A peak at ~323 °C is observed in all samples, corresponding to LDH dehydroxylation. Additional signals between ~290–375 °C and ~400–460 °C arise after functionalization and conjugation. The increased complexity of the deconvoluted profiles reflects progressive incorporation of silane and biomolecular species. The profiles correspond to individual measurements.

Table S1. Langmuir isotherm parameters for the adsorption of model proteins (BSA and α-CHY) on pristine and surface-functionalized LDH systems. The maximum adsorption capacity (Q_m) and the Langmuir constant (K_L) were determined from nonlinear fitting. Fitting parameters are presented as estimates ± standard errors. R^2 represents the coefficient of determination for each fit.

Particle	Protein	Q_m (μg/μg)	K_L (μg/ml)	R^2
LDH	BSA	1.21 ± 0.02	25.79 ± 2.44	0.998
LDH–NH_2	BSA	0.55 ± 0.02	14.37 ± 6.72	0.974
LDH–COOH	BSA	0.22 ± 0.04	161.13 ± 82.50	0.879
LDH	α-CHY	0.72 ± 0.05	149.21 ± 18.68	0.997
LDH–NH_2	α-CHY	0.47 ± 0.04	110.58 ± 19.32	0.992
LDH–COOH	α-CHY	0.76 ± 0.17	80.61 ± 43.21	0.906

Table S2. Freundlich isotherm parameters for the adsorption of rpZDIII on pristine and surface-functionalized LDH systems. Fitting parameters are reported as estimates ± standard errors (SE) obtained from nonlinear regression. R^2 represents the coefficient of determination for each fit.

Particle	K ($\mu g \cdot mL^n/\mu g^{n+1}$)	n	R^2
LDH	$9.77\times10^{-7} \pm 6.30\times10^{-7}$	4.63 ± 0.25	0.978
LDH–NH_2	$9.26\times10^{-5} \pm 7.14\times10^{-5}$	2.89 ± 0.28	0.952
LDH–COOH	$4.04\times10^{-6} \pm 7.01\times10^{-6}$	2.73 ± 0.40	0.975

Table S3. Physicochemical characterization of pristine LDH and rpZDIII-loaded LDH systems at different particle-to-protein ratios, including hydrodynamic size, polydispersity index (PDI), and ζ potential. Hydrodynamic size and ζ potential are reported as average ± standard deviation (SD, n = 3).

Sample/Label	Ratio	Size (nm)	SD (nm)	PDI	ζ (mV)	SD (mV)
LDH	–	70.33	1.72	0.133	50.53	5.65
LDH **A**	1:0.125	219.80	24.18	0.207	−8.24	1.52
LDH **B**	1:0.25	288.30	7.35	0.248	−11.78	3.63
LDH **C**	1:0.5	213.00	5.94	0.170	−15.54	2.06
LDH **D**	1:1	266.55	8.98	0.141	−16.61	1.26
LDH **E**	1:2	197.17	1.42	0.088	−20.33	2.04

Table S4. Physicochemical characterization of amine-functionalized LDH and rpZDIII-loaded LDH–NH_2 systems at different particle-to-protein ratios, including hydrodynamic size, polydispersity index (PDI), and ζ potential. Hydrodynamic size and ζ potential are reported as average ± standard deviation (SD, n = 3).

Sample/Label	Ratio	Size (nm)	SD (nm)	PDI	ζ (mV)	SD (mV)
LDH-NH_2	–	109.47	6.57	0.073	36.63	1.30
LDH-NH_2 **A**	1:0.125	137.37	2.58	0.194	−11.31	3.47
LDH-NH_2 **B**	1:0.25	172.83	7.38	0.120	−13.47	0.54
LDH-NH_2 **C**	1:0.5	166.77	5.84	0.124	−19.15	2.96
LDH-NH_2 **D**	1:1	176.87	2.70	0.147	−20.24	2.72
LDH-NH_2 **E**	1:2	161.17	12.81	0.139	−21.90	3.10

Table S5. Physicochemical characterization of carboxyl-functionalized LDH and rpZDIII-loaded LDH–COOH systems at different particle-to-protein ratios, including hydrodynamic size, polydispersity index (PDI), and ζ potential. Hydrodynamic size and ζ potential are reported as average ± standard deviation (SD, n = 3).

Sample/Label	Ratio	Size (nm)	SD (nm)	PDI	ζ (mV)	SD (mV)
LDH-COOH	–	152.43	5.27	0.092	−23.97	3.32
LDH-COOH **A**	1:0.125	148.83	3.55	0.107	−12.32	6.54
LDH-COOH **B**	1:0.25	157.43	12.25	0.118	−16.38	1.98
LDH-COOH **C**	1:0.5	161.67	2.80	0.134	−19.61	3.04
LDH-COOH **D**	1:1	164.27	9.72	0.161	−21.96	1.38
LDH-COOH **E**	1:2	146.57	4.01	0.098	−19.09	1.88

Calculating the amount of reactant to functionalize nanoparticles in suspension

Considering the concentration of nanoparticles (NP) (known after drying and weighing fixed volumes of NP suspensions), their geometric dimensions (by measuring individual NP from TEM images), and the reported material density, it is feasible to estimate the NP concentration and their surface area.

For a suspension of 1 mg/mL of Mg-Al layered double hydroxides (LDH) NP, visualized in TEM as hexagonal platelets with an average circumradius of 35 nm and a thickness of 5 nm (from more than 100 LDH NP counted, as the ones seen in Figure S1), and an average density of 2.06 g/mL reported for hydrotalcite-like structures, the calculated concentration is 3.05×10^{13} LDH NP per mL, equivalent to 50.65 nM, with a surface area of 7415.29 nm^2 per each LDH NP.

Considering the density and molecular weight of the reactant to functionalize the NP, it is feasible to estimate the circular area that each spherical molecule will occupy on a surface, and thus the amount required to cover the NP with one monolayer of molecules in a hexagonal packing arrangement.

For (3-aminopropyl)triethoxysilane (APTES) of 0.946 g/mL and 221.37 g/mol, the estimated area per molecule is 0.64 nm^2, which for the LDH NP described above results in 1.05×10^4 molecules per NP at the densest packing density of 0.907 from equal circular areas arranged in a hexagonal lattice. This estimate is equivalent to 0.53 mM of APTES that must be available in the suspension of 1 mg/mL of LDH NP to theoretically form one monolayer of APTES molecules on every LDH NP. APTES 50× in excess means having 26.5 mM readily in the suspension and thus adding 6.2 μL of the reagent.

Considering grafting densities reported for APTES, ranging from 0.5 to 2.5 molecules per nm^2, the calculated above corresponds to 1.56 molecules/nm^2. This estimate is comparable to the experimental values reported for boehmite of 14 nm, smaller than LDH but having similar octahedral networks of aluminum coordinated with hydroxyl groups, quantifying 1.2 and 1.7 molecules per nm^2, respectively, by TGA and EA [107]. Moreover, comparing APTES-modified NP suspensions of 2 mg/ml they quantified approximately 0.584 mM of amino groups with ninhydrin, while we report 0.597 ± 0.039 mM.

References

107. Zarinwall, A.; Waniek, T.; Saadat, R.; Braun, U.; Sturm, H.; Garnweitner, G. Comprehensive characterization of APTES surface modifications of hydrous boehmite nanoparticles. *Langmuir* **2021**, *37*, 171–179. https://doi.org/10.1021/acs.langmuir.0c02682.

116. Mokhtar, M.; Inayat, A.; Ofili, J.; Schwieger, W. Thermal decomposition, gas phase hydration and liquid phase reconstruction in the system Mg/Al hydrotalcite/mixed oxide: A comparative study. *Appl. Clay Sci.* **2010**, *50*, 176–181. https://doi.org/10.1016/j.clay.2010.07.019.

117. Zhang, X.; Wu, G.; Peng, X.; Li, L.; Feng, H.; Gao, B.; Huo, K.; Chu, P.K. Mitigation of corrosion on magnesium alloy by predesigned surface corrosion. *Sci. Rep.* **2015**, *5*, 17399. https://doi.org/10.1038/srep17399.

118. Lin, C.H.; Chu, H.L.; Hwang, W.S.; Wang, M.C.; Ko, H.H. Synthesis and optical properties of Mg-Al layered double hydroxides precursor powders. *AIP Adv.* **2017**, *7*, 125005. https://doi.org/10.1063/1.4990832.

119. Reed, T.; Wonderling, N.; Gevaudan, J.P.; Mauro, J.C. Effect of relative humidity on the interlayer spacing of phosphate intercalated Mg, Al layered double hydroxide (hydrotalcite-like) crystals. *J. Am. Ceram. Soc.*, **2025**, *108*, e20178. https://doi.org/10.1111/jace.20178.
